# Eggs of *Schistosoma japonicum* deposited in the spleen induce apoptosis of splenic T cells in C57BL/6 mice

**DOI:** 10.1007/s00436-025-08474-4

**Published:** 2025-03-10

**Authors:** Yanjuan Wang, Yuan Hu, Jing Zhang, Danling Zhou, Yanjun Zhang, Jianping Cao

**Affiliations:** 1 Shanghai Urban Construction Vocational College, Shanghai, 201415 China; 2 National Key Laboratory of Intelligent Tracking and Forecasting for Infectious Diseases; Key Laboratory of Parasite and Vector Biology, National Health Commission of the People’s Republic of China; National Institute of Parasitic Diseases at Chinese Center for Disease Control and Prevention (Chinese Center for Tropical Diseases Research); World Health Organization Collaborating Centre for Tropical Diseases, Shanghai, 200025 China

**Keywords:** *Schistosoma japonicum*, Apoptosis, Spleen, Egg granulomas, Lymphocytes

## Abstract

To explore the relationship between *Schistosoma japonicum* egg deposition and the resultant structural damage to the spleen, mice were infected percutaneously with cercariae or eggs were surgically injected into their spleens. Terminal transferase dUTP nick-end-labeling (TUNEL) showed that cells around the *S. japonicum* eggs were apoptotic in vivo. Flow cytometry revealed a sharp reduction in splenic B and T cells at 8 weeks post-infection (p.i.) and a significant increase in Annexin V positive T cells. Immunochemistry showed that the remaining follicles in the spleen at 16 weeks p.i. comprised mainly B lymphocytes. Comparing T lymphocytes in the spleen and liver egg granulomas showed obvious CD3^+^ positive areas in the spleen, indicating that splenic egg granulomas have a different cellular composition to liver granulomas. *S. japonicum* eggs deposited in the spleen might induce apoptosis of splenic cells, especially T lymphocytes. When splenic lymphocytes were cultured in vitro with *S. japonicum* soluble egg antigen (SEA), more cells underwent apoptosis at an antigen concentration of 120 μg/ml compared to 60 μg/ml at all times p.i.. Cells from 8 weeks p.i. seemed more susceptible to SEA-induced apoptosis. Further research should be focus on the molecule(s) that induce T cells apoptosis, which might provide clues to the mechanisms of immunosuppression during *S. japonicum* infection and will promote vaccine research.

**Supplementary Information:**

The online version contains supplementary material available at 10.1007/s00436-025-08474-4.

## Introduction

Schistosomiasis is a neglected tropical disease affecting more than 200 million people worldwide (Siqueira et al. 2017). Schistosome eggs can induce the formation of inflammatory granulomas in the liver, intestine, lung, urinary bladder, even in skin and the spinal cord (Ross et al. 2012). The spleen was observed to have the ability to limit the formation of CD4^+^ T cell-mediated granulomas (Garb et al. 1981; Kayes and Colley 1979).

Our previous study showed that *S. japonicum* infection destroys the structure of the splenic lymphoid follicles in mice (Ji et al. 2008). Further examination of spleen homogenates from 5 weeks p.i., the earliest time at which egg deposition in the liver can be observed, showed that egg deposition in the spleen began at 7 weeks p.i. and increased gradually as the infection progressed (Wang et al. 2015). However, interestingly, not all the infected mice exhibited the same spleen damage, with some lymphoid follicles within the granulomatous spleens maintaining their structural integrity until 20 weeks p.i., unlike the lymphoid follicles in spleens without egg granulomas.

To explore the relationship between egg deposition and the resultant structural damage to the spleen, we detected the apoptosis of spleen lymphocytes. Indeed, many studies have suggested that antigens of *Schistosoma mansoni* can induce apoptosis of host T cells (Carneiro-Santos et al. 2000; Chen et al. 2002), and this selective apoptosis contributes to the immunosuppression associated with chronic schistosomiasis (Rumbley et al. 2001).

## Materials and methods

### Ethics statement

All animal experiments were performed in strict accordance with the Regulations for the Administration of Affairs Concerning Experimental Animals of China, and efforts were made to minimize animal suffering. All procedures performed on animals in this study were approved by the Laboratory Animal Welfare & Ethics Committee of the National Institute of Parasitic Diseases at Chinese Center for Disease Control and Prevention (Chinese Center for Tropical Diseases Research; approval ID: IPD 2019–12).

### Animals and parasites

C57BL/6 mice (6–8 weeks old) were obtained from the Shanghai Laboratory Animal Center (Shanghai, China). *Oncomelania hupensis* snails harboring *S. japonicum* cercariae were purchased from the Jiangsu Institute of Parasitic Diseases (Wuxi, China).

### *S. japonicum* infection and immunization

Mice were infected percutaneously with 20 cercariae of *S. japonicum* and sacrificed humanely at various intervals after infection. *S. japonicum* SEA was prepared as described previously (Wang et al. 2008). Normal mice were immunized by subcutaneously injection with 50 µg of SEA in complete Freund's adjuvant (Sigma, USA). The apoptotic response of T cells to SEA was measured at day 10 post immunization.

### Injection of eggs

Injection of eggs into mouse spleens was carried out as described previously (Botros et al. 1986) with minor modifications. Briefly, mice were anesthetized by intraperitoneal injection of pentobarbital, their left flanks were shaved, and the skin was washed with 75% ethanol. A 5–8 mm incision was made in the skin just below and parallel to the lowest rib using a scalpel. The spleen was gently lifted out and 0.1 ml of a suspension of about 2500 eggs was injected into the lower pole of the spleen using a 3–4 mm needle. Afterwards, the peritoneum and skin were sutured. The eggs used for injection were isolated under sterile conditions from the livers of mice at 6 weeks p.i. and used when alive. Mice injected with freeze-dried eggs or saline solution were used as controls.

### Spleen histopathology and immunohistochemistry

Mice were sacrificed humanely, and their spleens were removed. The spleens were embedded in paraffin, sectioned, and stained with hematoxylin and eosin (H&E) prior to microscopic examination. To observe normal splenic B or T lymphocytes in follicles, rat anti-B220 (protein tyrosine phosphatase receptor type C) and anti-CD3 (both BD, USA) were used to detect B and T cells. Prepared sections were deparaffinized and repaired, and endogenous peroxidase was quenched by incubating the slides for 30 min in phosphate-buffered saline (PBS) with 0.3% H_2_O_2_. Binding of B220 was detected using a biotinylated anti-Ig horseradish peroxidase (HRP) detection Kit (BD), and binding of CD3 was detected using a streptavidin alkaline phosphatase (AP) detection kit (Dakewe, China). T or B cells in the livers of infected mice were detected using an HRP detection Kit (BD). All antibody incubations were conducted for 1 h at room temperature in a humidified box after dilution in PBS. The HRP activity was developed using diaminobenzidine (DAB). AP activity was developed using nitro blue tetrazolium/ 5-bromo-4-chloro-3-indolyl phosphate (BCIP).

### Analysis of apoptotic cells by TUNEL staining

Apoptotic cells around the eggs in the spleen of infected mice and injected mice were evaluated using a TUNEL assay kit (Calbiochem, Germany) following the supplier’s guidelines. Following permeabilization with proteinase K for 20 min, paraffin-embedded spleen sections were deparaffinized and rehydrated, and then incubated with a mixture of fluorescein isothiocyanate (FITC)-labeled deoxynucleotides and terminal deoxynucleotidyl transferase (TdT enzyme) for 1 h at 37 °C. Apoptotic cells in pooled splenocytes from infected mice at different times p.i. were detected using the same kit following the manufacturer’s instructions. Apoptotic cells labeled using the TUNEL assay kit were analyzed using flow cytometry.

### Cell preparation and flow cytometry

Splenocytes were obtained from infected mice and single cell suspensions were filtered through a 70 µm Nytex membrane (BD). Following red blood cell lysis using Tris-NH_4_Cl, the cells were washed three times in Roswell Park Memorial Institute (RPMI)−1640 (Invitrogen, USA) and adjusted to 10^6^ cells/ml. Cell viability was always > 95%, as determined using the trypan blue exclusion method. To detect apoptosis of the lymphocytes cultured with SEA in vitro, cells were cultured at 10^6^ cells/ml in 24-well microtiter plates for 48 h, and then stained with anti-B220-Allophycocyanin and anti-CD3-Phycoerythrin (both Biolegend, USA) in combination with Annexin V-FITC (BD). Stained cells were analyzed using a FACS Calibur flow cytometer (BD).

### Statistical analysis

Data analysis was performed using SPSS 29.0 for Windows (IBM Corp., USA). One way-analysis of variance (ANOVA) was used to compare the apoptosis rates between two groups of cells. *P* values < 0.05 were considered to indicate statistical significance.

## Results

### Cells around eggs deposited in the splenic granulomas in infected mice undergo apoptosis

**Fig. 1:**
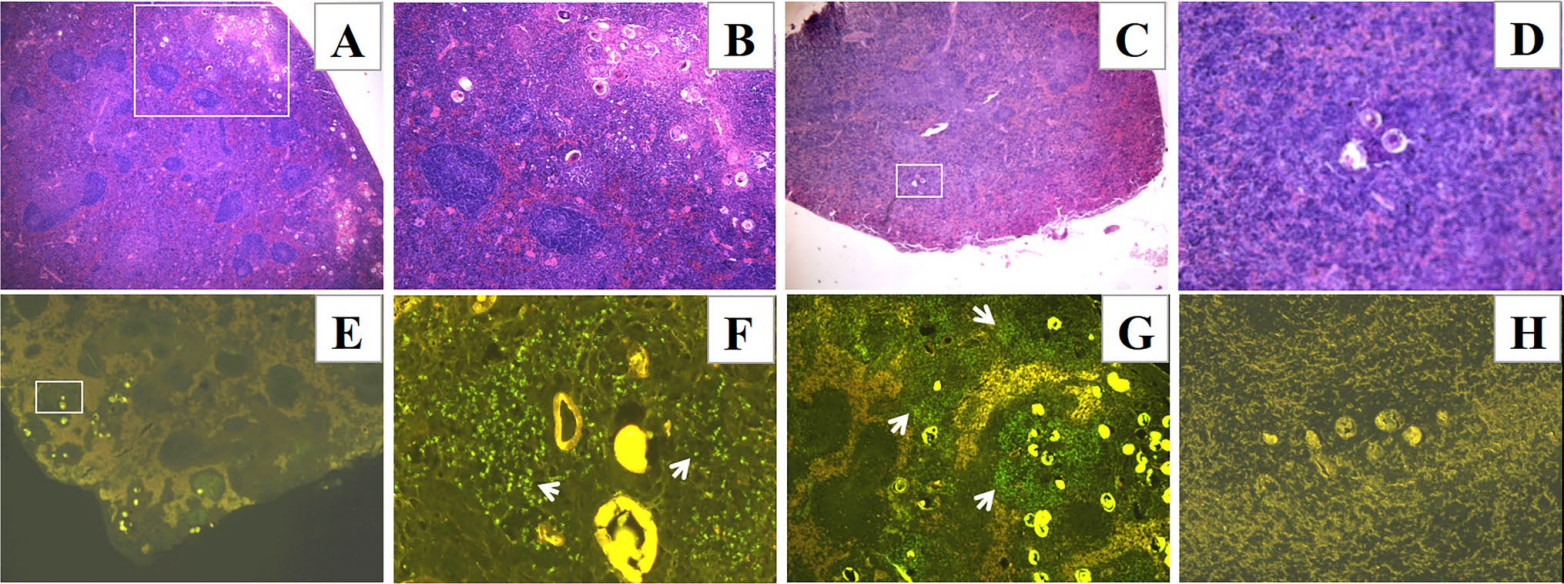
Cells around eggs deposited in the splenic granulomas undergo apoptosis. H&E staining of paraffin-embedded spleen sections prepared from C57BL/6 mice at 16 weeks p.i. (**A–D**). **A** Spleen sections with eggs deposited in granulomas (× 40), the area in the white rectangle is magnified in **B** (× 100). **C** Spleen sections without egg granulomas (× 40), the area in the white rectangle is magnified in **D** (× 200). Apoptotic cells were detected on spleen sections using TUNEL staining (FITC, green) (**E–H**). **E** Cells around eggs in the granulomas undergo apoptosis at 8 weeks p.i. (× 40), the rectangle area was magnified to show apoptotic cells (white arrows) in a granuloma in** F** (× 400). **G** Cells around eggs in the granulomas undergo apoptosis (white arrows) at 16 weeks p.i. (× 100). **H** Apoptotic cells were not detected on spleen sections without egg granulomas at 16 weeks p.i. (× 200). (*n* = 15 for mice at 16 weeks p.i.; *n* = 5 for mice at 8 weeks p.i., each stain was applied once for each mouse, and representative staining is shown)

Our previous work showed that *S. japonicum* eggs were found in spleen homogenates, with the structure of the splenic lymphoid follicles being destroyed (Wang et al. 2015). To evaluate whether the splenic cells around the deposited eggs were apoptotic, we examined the spleen histopathology and TUNEL staining was applied to detect apoptotic cells in the spleen with eggs deposition. The results showed that eggs were commonly deposited in the spleen of C57BL/6 mice as the infection progressed and usually led to the formation of granulomas (Fig. 1A, B), only in rare cases did the eggs deposited in the spleen not cause inflammatory cell aggregation, even at 16 weeks p.i. (Fig. 1C, D). Apoptotic cells around the eggs in the granulomas were detected in the spleens of infected mice (Fig. 1E–G), but were not detected on the sections without granulomas (Fig. 1H).

### Cells around eggs injected into the spleen undergo apoptosis

**Fig. 2:**
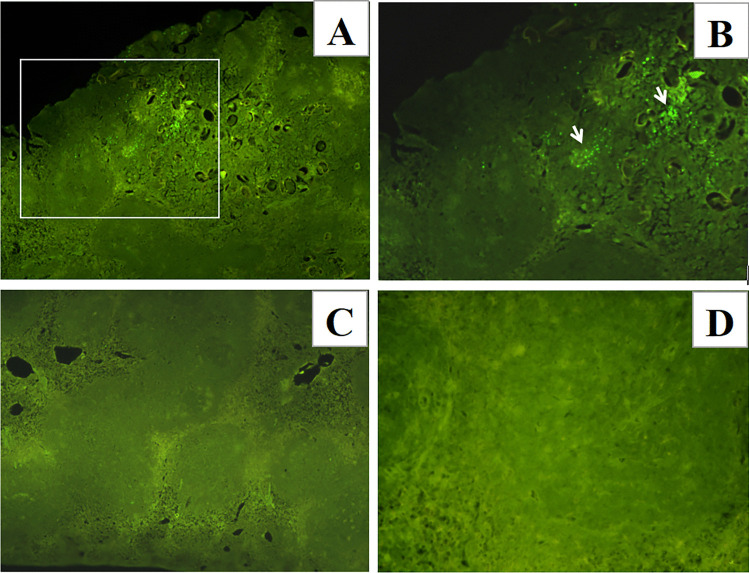
Cells around eggs injected in the spleen undergo apoptosis. Paraffin-embedded spleen sections prepared from C57BL/6 mice at 48 h after injection (**A–D**). **A** Apoptotic cells around fresh-eggs detected using TUNEL staining (FITC, green) (× 100), the area in the white rectangle is magnified in **B** (× 200) to show the location of apoptotic cells (white arrows). Apoptotic cells were not detected on spleen sections from mice injected with freeze-dried eggs in **C** (× 100) or saline solution in **D** (× 200). (*n* = 5 for each group of mice, each stain was applied once for each mouse, and representative staining is shown)

To test if *S. japonicum* eggs could induce apoptosis of splenic cells, TUNEL staining was also applied to detect apoptotic cells in egg-injected spleens. Apoptotic cells were detected around fresh-eggs at up to 48 h after injection (Fig. 2A, B), but could not be detected on the sections from spleens injected with freeze dried eggs (Fig. 2C) or saline injected mice (Fig. 2D).

### Splenic T cells are susceptible to apoptosis

In a previous study, we found that some lymphoid follicles within the granulomatous spleens maintained their structural integrity until 20 weeks p.i., which were not completely destroyed or were re-established, unlike the lymphoid follicles in spleens without egg granulomas (Wang et al. 2015).

**Fig. 3:**
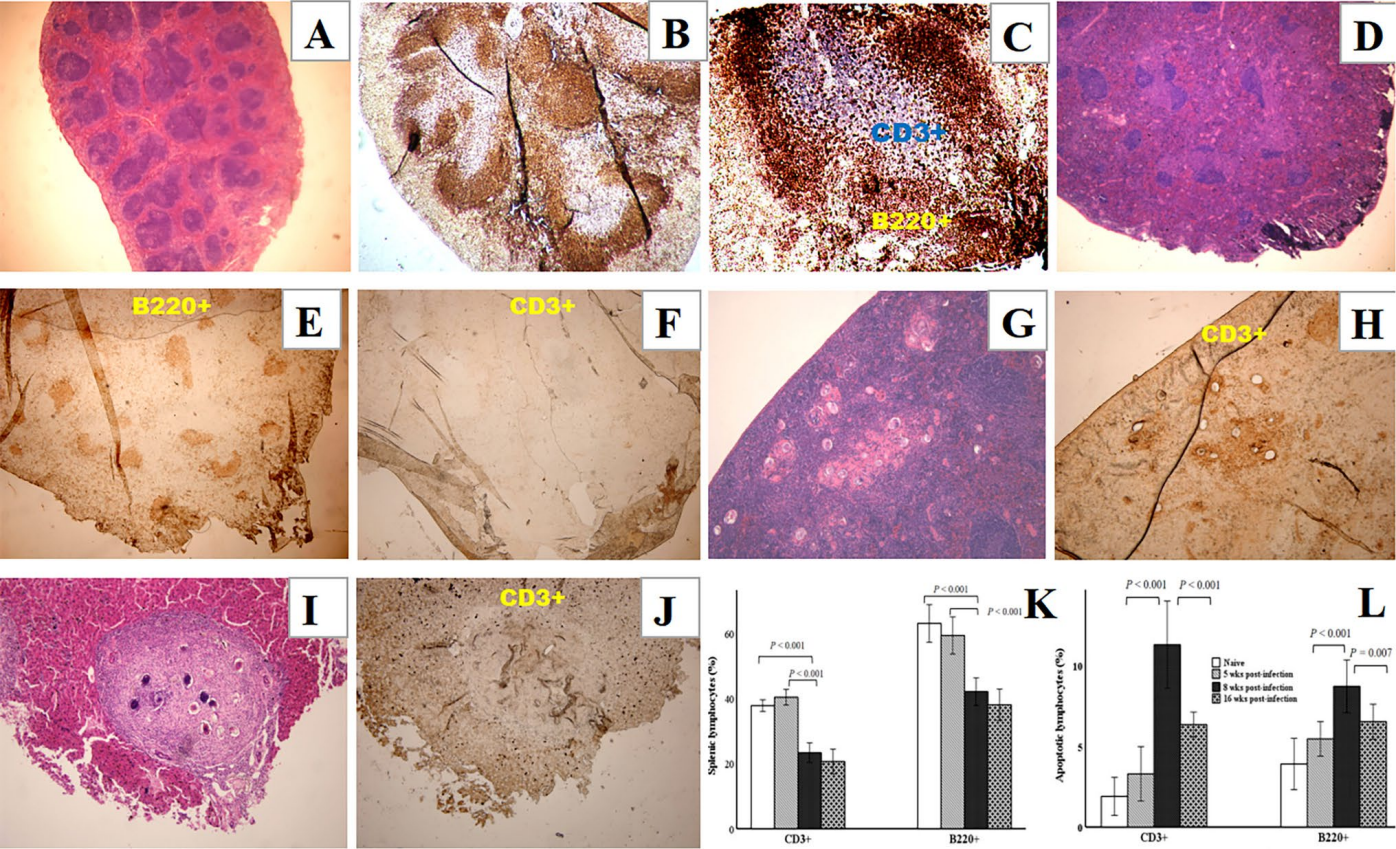
Splenic T cells of infected mice are susceptible to apoptosis. Spleen sections from naive mice with an intact lymphoid follicular structure are shown in **A–C**. **A** Sections stained with H&E (× 40), **B** and** C** Sections stained with anti-B220 (red) to detect B cells, and with anti-CD3 (blue) to detect T cells (B, × 40; C, × 100). Spleen sections from mice at 16 weeks p.i. are shown in **D–F**. **D** Sections stained with H&E (× 40); **E** and **F** Sections stained with anti-B220 or anti-CD3 to detect B or T cells (red) in the same spleen (E, × 40; F, × 40). H&E and immunohistochemical (IHC) staining of both the spleen and liver from mice at 16 weeks p.i. are shown in **G–J**. Sections from the spleen and liver stained with H&E, and CD3 staining in different slides of the same samples showing the T cells (red). **G** and** H** Sections of spleen (G, H&E × 100; H, IHC × 100). **I** and** J** Sections of liver (I, H&E × 100; J, IHC × 100). (*n* = 15 for mice at 16 weeks p.i., *n* = 5 for groups of naive mice (each stain was applied once for each mouse, and representative staining is shown). **K** and **L** Splenic cells from naive or infected mice were stained with anti-B220 and anti-CD3 antibodies in combination with Annexin V. **K** B220^+^ or CD3^+^ cells in the spleens of mice at different times p.i. were analyzed using flow cytometry. **L** Apoptotic splenic B220^+^ or CD3.^+^ cells were also examined. (*n* = 5 for each group of mice. Each sample was tested once, and the results are presented as the mean ± SD. Statistically significant differences between the compared groups are shown as *p* values)

In this study, we examined the cellular composition of the follicles in the spleens of mice at 16 weeks p.i., which differed from the integrated follicular structure and distinct B and T zones observed in the spleens of uninfected mice (Fig. 3A–C). The cells left in the splenic follicles of infected mice comprised mostly B lymphocytes, with no T lymphocytes being detected (Fig. 3D–F). However, T cells were detected around the eggs in the granulomas in spleen at the same time (Fig. 3G, H), which also differed from the cellular composition of liver granulomas (Fig. 3I, J). Flow cytometry revealed consistent results: a sharp reduction of splenic B and T cells and a significant increase of Annexin V positive T cells at 8 weeks and 16 weeks p.i. (Fig. 3K, L). These results suggested that T lymphocytes were gathered around the eggs in spleen granulomas and underwent apoptosis.

### SEA could induce apoptosis of splenic T lymphocytes in a concentration-dependent manner

**Fig. 4:**
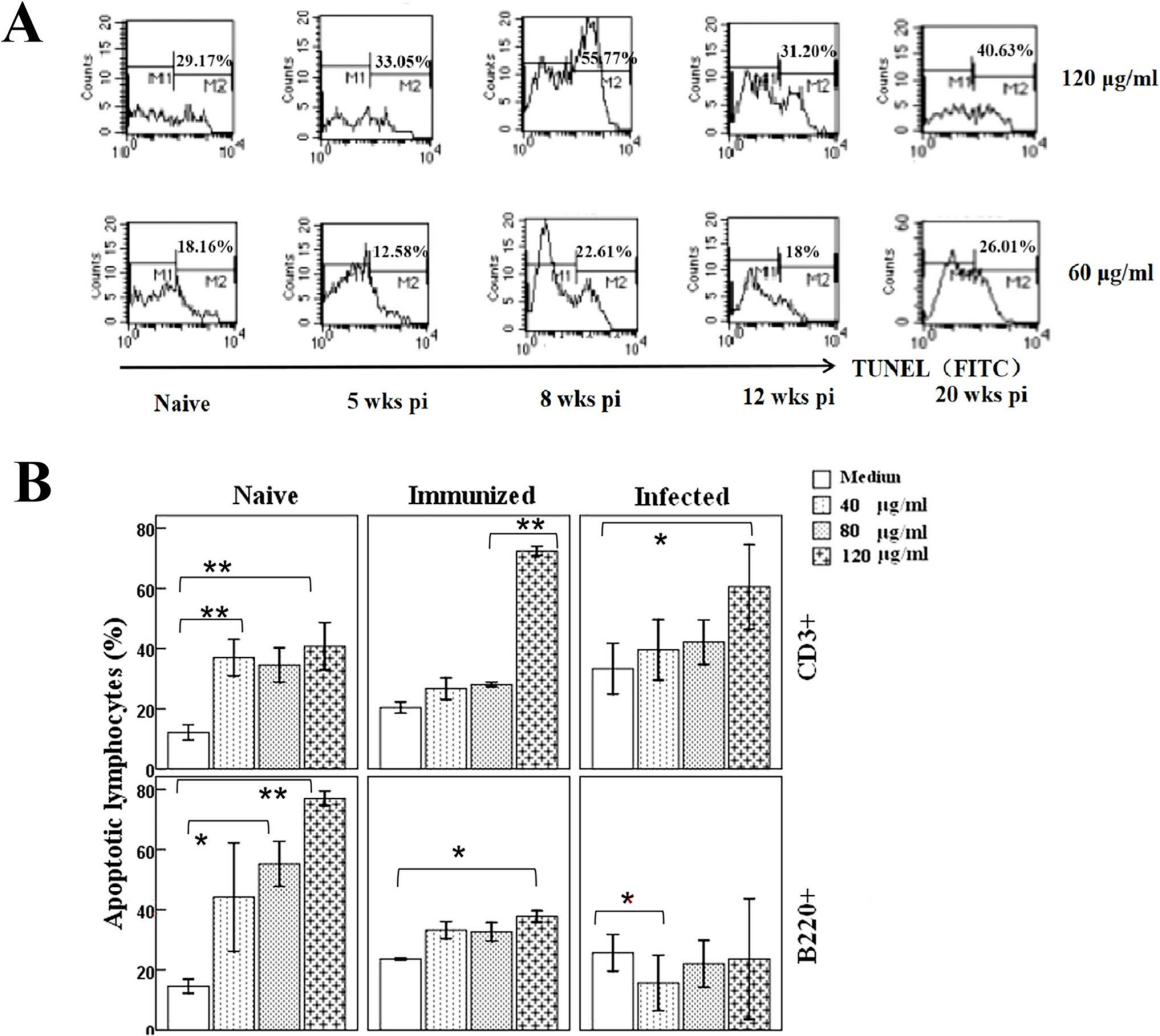
Splenic T lymphocytes undergo apoptosis in the presence of a high concentration of SEA in vitro. **A** Splenic cells of infected mice were pooled from three to four animals at each time point. (*n* = 4 for naive mice and mice at 5 weeks p.i., *n* = 3 for mice at 8, 12, and 20 weeks p.i.). Cells were cultured at 10^6^ cells/ml in 24-well microtiter plates for 48 h in the presence of SEA (60 μg/ml and 120 μg/ml). Apoptotic cells labeled by FITC-deoxynucleotides were analyzed using flow cytometry (one sample at each concentration). **B** Splenic lymphocytes from naive mice, immunized mice, and mice at 8 weeks p.i. (*n* = 4 mice per group) were cultured at 10^6^ cells/ml for 48 h in the presence of SEA (40 μg/ml, 80 μg/ml, and 120 μg/ml), apoptotic B220^+^ or CD3^+^ cells labeled with Annexin V were analyzed by flow cytometry. Each sample was tested once, and the results are presented as the mean ± SD (* *P* < 0.05, ** *P* < 0.001)

To investigate the relationship between *S. japonicum* eggs and splenic T cell apoptosis, splenic lymphocytes were cultured with SEA in vitro. More cells underwent apoptosis at an SEA concentration of 120 μg/ml compared to 60 μg/ml at all times p.i. (Fig. 4A). Cells from mice of 8 weeks p.i. seemed to be more susceptible to SEA-induced apoptosis; therefore, we analyzed the pro-apoptotic activity of SEA in splenic lymphocytes from naive mice, immunized mice and mice of 8 weeks p.i., the results suggested that as the concentration of SEA increased, the number of apoptotic T cells became markedly higher than that of B cells (Fig. 4B), especially in the cells from immunized and infected mice.

## Discussion

During the course of *S. japonicum* infection, parasite eggs are trapped in the liver of their vertebrate host, followed by the development of granulomas. The granulomas obstruct portal circulation, leading to portal hypertension, after which the spleen become markedly enlarged from six weeks p.i.. However, the pathology of the spleen, as one of the major organs affected during schistosomiasis, has long been overlooked.

Our previous study showed that *S. japonicum* infection destroys the structure of the splenic lymphoid follicles in mice, which would lead to a reduced immune response during late infection stages (Ji et al. 2008). Subsequently, we observed egg deposition in the spleen of infected mice and focused on the splenic pathology, showing that deposition of schistosome eggs in the spleen was a common phenomenon, which could change the microenvironment and morphology of the spleen as the infection progressed (Wang et al. 2015). Herein, to observe the destructive effect of the eggs on the spleen, we assessed the pro-apoptotic activity of *S. japonicum* SEA on splenic lymphocytes.

The ability of schistosomes to induce apoptosis in host immune cells was established in murine and human schistosomiasis (Carneiro-Santos et al. 2000). In the present study, apoptotic cells were found in the granulomas around the eggs in mouse spleens, which is consistent with the view that cells in the granulomas are susceptible to apoptosis, because lymphocytes are more highly activated and their cell division ability is inhibited in granulomas (Rumbley et al. 1998). Research also demonstrated that lymphocytes activated by SEA initially appear in the spleen, and then migrate into the granulomas, where they become fully activated by high antigen concentrations (King et al. 2001). These findings are compatible with a previous in vitro study which suggested that as the concentration of SEA gradually increased, the proliferation of splenocytes was inhibited and apoptosis rates increased (Rumbley et al. 2001). These findings might explain why apoptosis was only observed in cells around eggs in granulomas.

Apoptotic stimuli comprise extrinsic and intrinsic signals (Dash et al. 2007). Extrinsic signals consist of the binding of death-inducing ligands, including the Fas ligand, tumor necrosis factor alpha (TNFα), and TNF-related apoptosis inducing ligand (TRAIL), and most studies have focused on the pathway through which T cells undergo apoptosis (Qi et al. 2012). Intrinsic signals are usually caused by cellular stress, such as growth factor deprivation. Splenic lymphocytes appear to undergo apoptosis through both the extrinsic and intrinsic pathways (Rumbley et al. 1998; Rutitzky et al. 2003). In our study, apoptosis was also observed around the site of eggs injection, indicating a relationship between the antigens excreted from the eggs and apoptosis. The distribution of SEA-derived proteins within macrophages was detected in the marginal zones of the spleen from 4 weeks p.i. (El-Dosoky et al. 1984). In addition, SEA has effects related to the downregulation of the host immune response in chronic schistosomiasis, including an increase in Forkhead box-P3 (Foxp3)^+^ regulatory T cells (Tregs) (Zaccone et al. 2010, 2009), and apoptosis of other types of T cells. A study reported apoptosis of both CD4^+^ and CD8^+^ T cells in mice (Estaquier et al. 1997), while another study revealed higher apoptosis rates in CD8^+^ T cells (Fallon et al. 1998), which contrasted with findings showing that CD4^+^, but not CD8^+^ T cells undergo apoptosis in infected C57BL/6 mice (Rutitzky et al. 2003). B lymphocytes were supposed to act as Fas L-bearing mediators, because depletion of B cells resulted in decreased CD4^+^ T cell apoptosis (Lundy et al. 2001; Lundy and Boros 2002). Our data showed a sharp reduction of splenic B and T cells at 8 weeks p.i., with T cells demonstrating a significant increase in apoptosis. Considering our previous findings that lymphoid follicles within the granulomatous spleens maintained their structural integrity in late infection (Wang et al. 2015), we examined the cellular composition of follicles in the spleens of mice at 16 weeks p.i. The results indicated that the cells remaining in the splenic follicles of infected mice comprised mostly B lymphocytes, with no T lymphocytes. By contrast, T cells were detected around the eggs in spleen granulomas, but not in liver granulomas. These results suggested a relationship between the eggs deposited in splenic granulomas and apoptosis of splenic T lymphocytes. Our results also suggested that splenic T cells are involved in the formation of splenic egg granulomas in mice during late infection; therefore, T cell apoptosis in granulomas might be the reason for the absence of T zones in the lymphoid follicles in the spleens of infected mice.

Schistosome granulomas isolated from different organs differ dramatically in their cell composition (Weinstock and Boros 1983). Liver granulomas comprise 50% eosinophils and show the highest percentage of lymphocytes, while in contrast, ileum granulomas consist of 75% macrophages, with no lymphocytes. The observation of T cell areas around splenic granulomas, even at 16 weeks p.i., partially supported our assumption that splenic egg granulomas have a different cellular composition to liver granulomas. This phenomenon might occur because the spleen is an important immune organ that is enriched with lymphocytes. For the T lymphocytes found in the liver granulomas, a possible explanation is that they represent an increased population of T cells that can alleviate granuloma pathology, including Tregs (Nation et al. 2022, 2020) and CD4^+^ T follicular helper (Tfh) cells (Yang et al. 2019), which might help to create a less immunologically hostile environment for the worms during late infection.

In this study, more cells underwent apoptosis at a high concentration of SEA in vitro, while apoptosis of cells at the concentrations below 60 μg/ml (Supplementary data sheet 1) was not obvious, which might have been influenced by antigen stimulated cell proliferation. A study reported that 6.25 μg/ml of SEA from *Schistosoma haematobium* was sufficient to stimulate cell proliferation and reduce apoptosis in cultured human urothelial cells (Botelho et al. 2013); however, in another recent study, the results suggested that SEA from *S. mansoni* can dose-dependently inhibit the growth and progression of colonic cancer cells (Pekkle Lam et al. 2024). The result can also be influenced by the limitations of the TUNEL assay itself, it was reported that both in cell culture and in vivo, the breaks in DNA were repaired rapidly if the NaCl concentration was lowered (Dmitrieva and Burg. 2007), which might be solved by adding other detection methods to increase the accuracy of experimental results.

Opinions on the contribution of apoptosis differ, with most studies presuming that it can limit the pathology as the infection progresses. Research has demonstrated a higher rate of CD4^+^ T cell apoptosis in granulomas and mesenteric lymph nodes from infected C57BL/6 mice compared with those from CBA mice (Rutitzky et al. 2003). Mice with few pathological symptoms exhibited enhanced CD4^+^ T cell apoptosis (Stadecker et al. 2004). In addition, a study reported that during the early stage of *S. japonicum* infection, non-egg antigens triggered T helper cell 2 (Th2) cell apoptosis, thus contributing to Th1 polarization. By contrast, egg antigens triggered Th1 cell apoptosis, leading to Th2 polarization (Xu et al. 2010). In addition to schistosomes, other helminths also induce apoptosis of host cells (Gazzinelli-Guimarães et al. 2013; Zepeda et al. 2017).

**Fig. 5:**
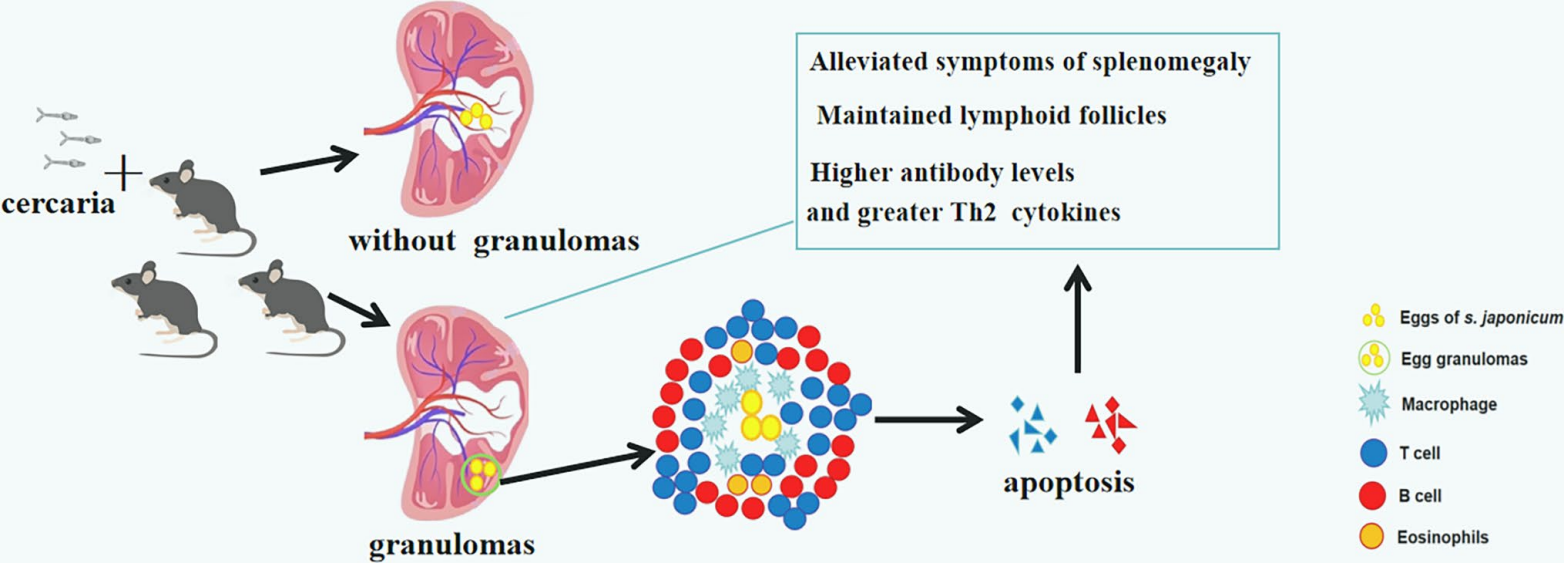
Schematic diagram of the hypothesis based on the results of the present study. Egg deposition in the spleens of infected mice occurred frequently, but only occasionally led to granuloma formation. A large number of macrophages, eosinophils, and lymphocytes gathered around the eggs. As the infection progressed, more T lymphocytes migrated into the granuloma area and underwent apoptosis in the presence of high concentrations of SEA in the granulomas, which might lead to alleviation of the symptoms of splenomegaly and an altered immune environment in the spleen

Granulomas are formed around parasite eggs to mitigate the cytotoxic effects of egg-secreted antigens. Egg-secreted antigens play a vital role in the course of infection, the Th1 immune response changes after egg laying, and becomes predominantly Th2. This shifting is essential to limit tissue damage and maintain the long-term balance between the chronically infected host and the long-lived parasite (Sanches et al. 2020). Based on the present study, we hypothesize that the T cell apoptosis caused by high concentration in granulomas might be the reason for the changing immune response in the spleen (Fig. 5). Mice with a granulomatous spleen showed more apoptotic cells in situ, alleviated symptoms of splenomegaly (Supplementary data sheet 2), higher antibody levels and more Th2 cytokines (Wang et al. 2015).

The SEA extract from schistosomes comprises hundreds to thousands of proteins, glycoproteins, and lipids. The SEA proteins are well recognized for their ability to stimulate a type 2 immune response (De Marco Verissimo et al. 2019). Proliferation of acute- and chronic-infection splenic T cell differed when treated with different fractions of SEA prepared by SDS-PAGE separation and electroelution (Lukacs and Boros 1991). In addition, fractionation and analysis of the molecules in the excretory-secretory products derived from *S. mansoni* suggested that the pro-apoptotic activity of skin-stage schistosomula was associated with a protein of ~ 23 kDa (Chen et al. 2002). In addition, a recombinantly expressed protein from *S. japonicum* was reported to induce apoptosis in murine myeloid leukemia cells (Yang et al. 2013). We hypothesized that as a complex mixture isolated from schistosome eggs, the protein inducers and inhibitors of apoptosis might co-exist in SEA. The identity of the molecule(s) with pro-apoptotic activity in SEA, and the mechanisms of the induction of apoptosis in splenic T lymphocytes, are the subjects of ongoing studies. We have already narrowed the range to a fraction of SEA molecules between 55 and 72 kDa (Wang and Cao 2023), and we believe the results will provide candidate molecules for future vaccine research.

## Supplementary Information

Below is the link to the electronic supplementary material.

Supplementary file1 (DOCX 12033 KB)

Supplementary file2 (DOCX 2520 KB)

Supplementary file3 (DOCX 466 KB)

Supplementary file4 (XLSX 13 KB)

## Data Availability

No datasets were generated or analysed during the current study.

## Abbreviations

TUNEL: Terminal transferase dUTP nick-end-labeling
p.i.: Post-infection
SEA: Soluble egg antigen
PBS: Phosphate-buffered saline
HRP: Horseradish peroxidase
AP: Alkaline phosphatase
DAB: Diaminobenzidine
SPSS: Statistical package for social science
ES: Excretory-secretory
