# Supplementary material for: Eggs of Schistosoma japonicum deposited in the spleen induce apoptosis of splenic T cells in C57BL/6 mice
Source: Parasitol Res. 2025 Mar 10;124(3):31. doi: 10.1007/s00436-025-08474-4 (PMC11891099; doi:10.1007/s00436-025-08474-4)
Supplement: Supplementary file 1 — Supplementary file1 (DOCX 12033 KB) [file 436_2025_8474_MOESM1_ESM.docx]

**
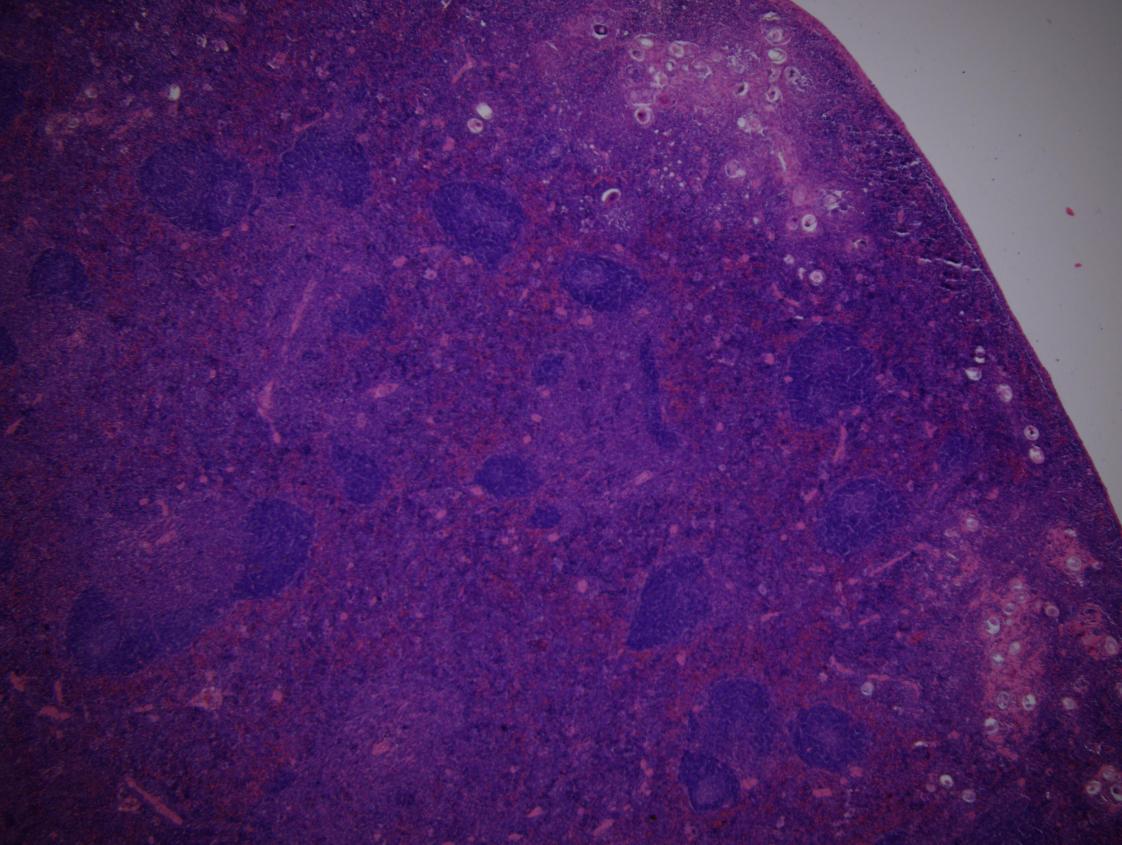
**

Figure 1B

Figure 1A

**
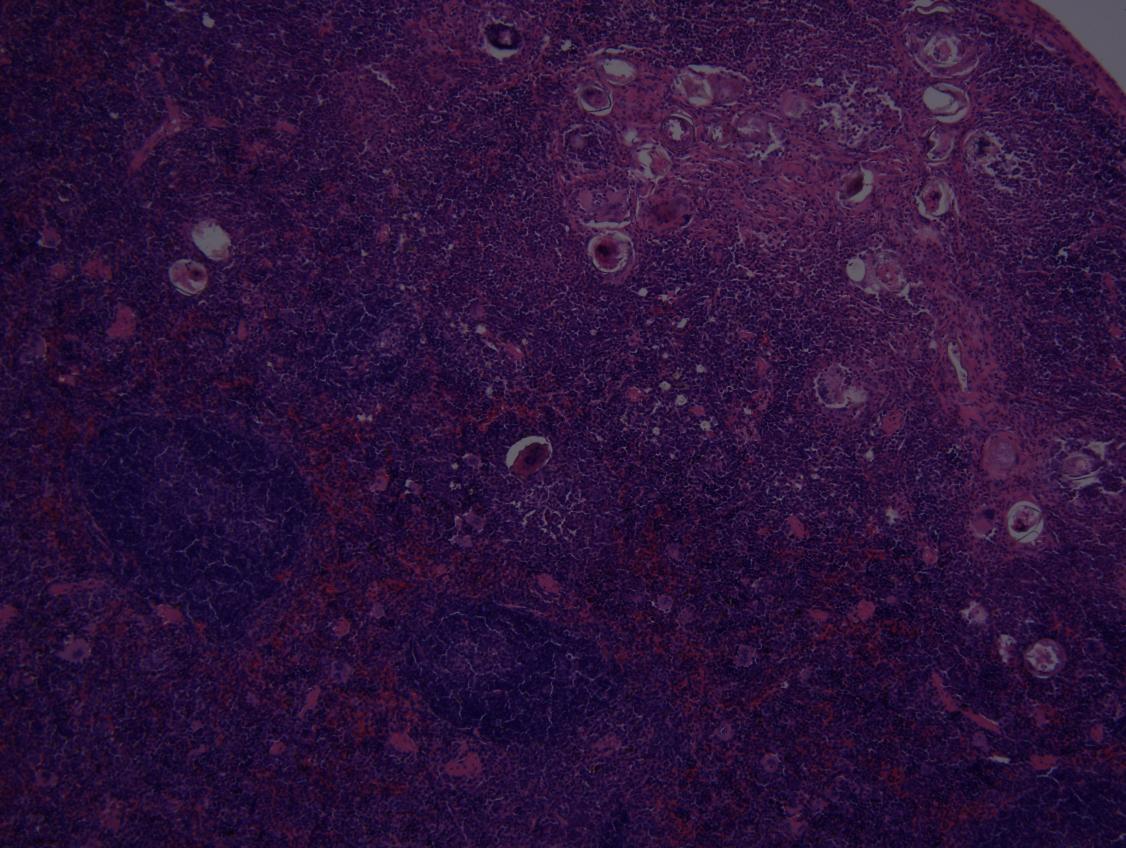
**

**
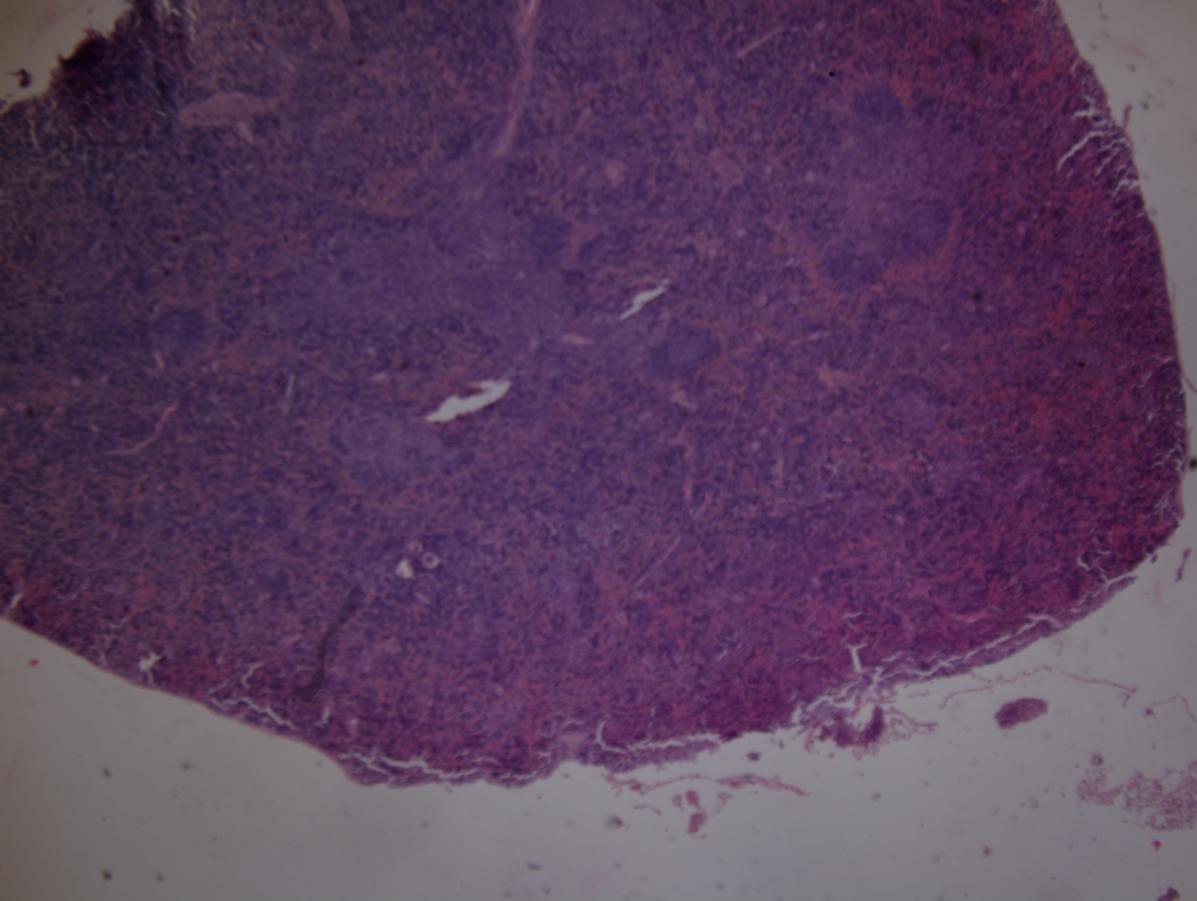
**

Figure 1D

Figure 1C

**
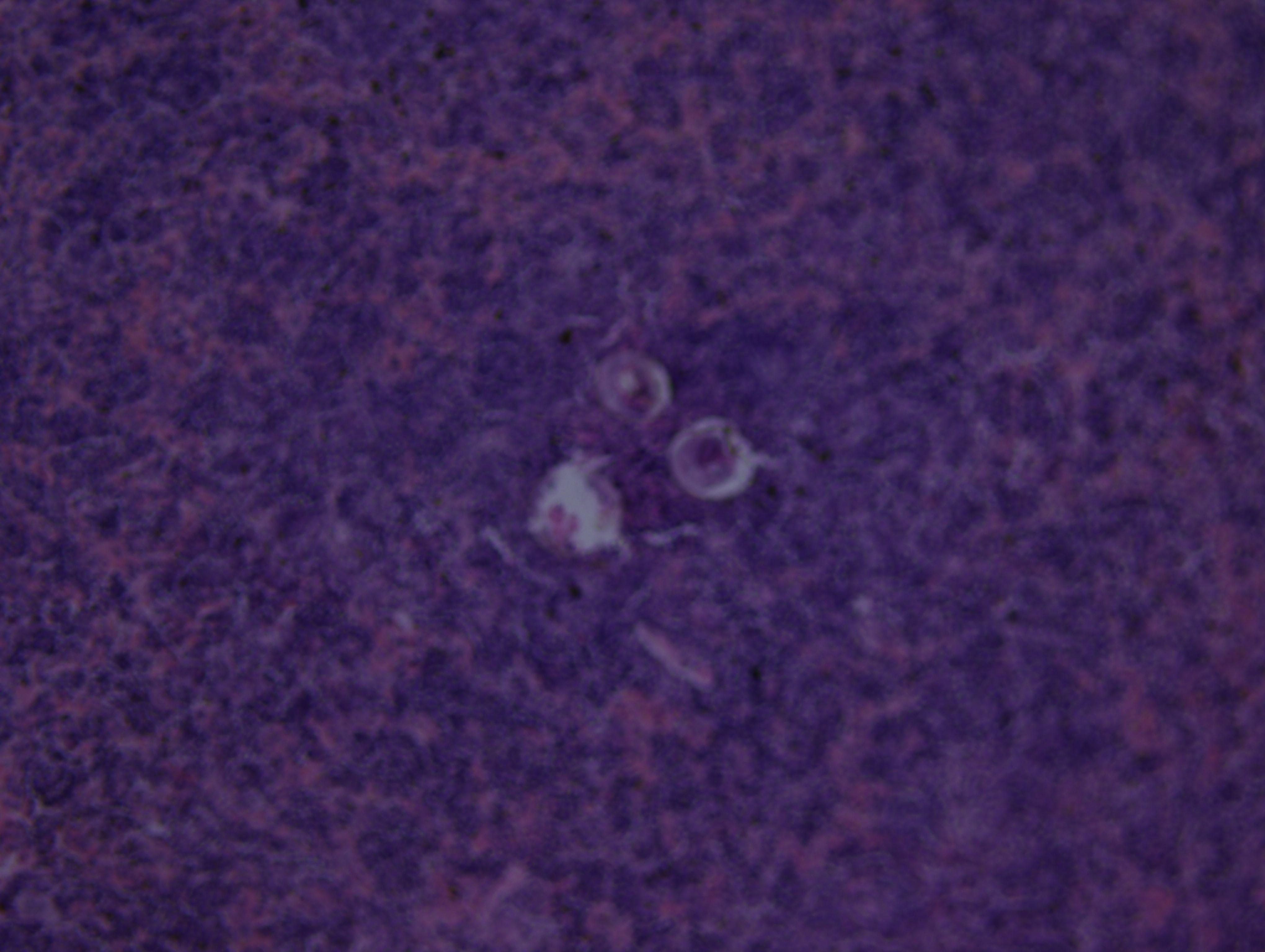
**

**
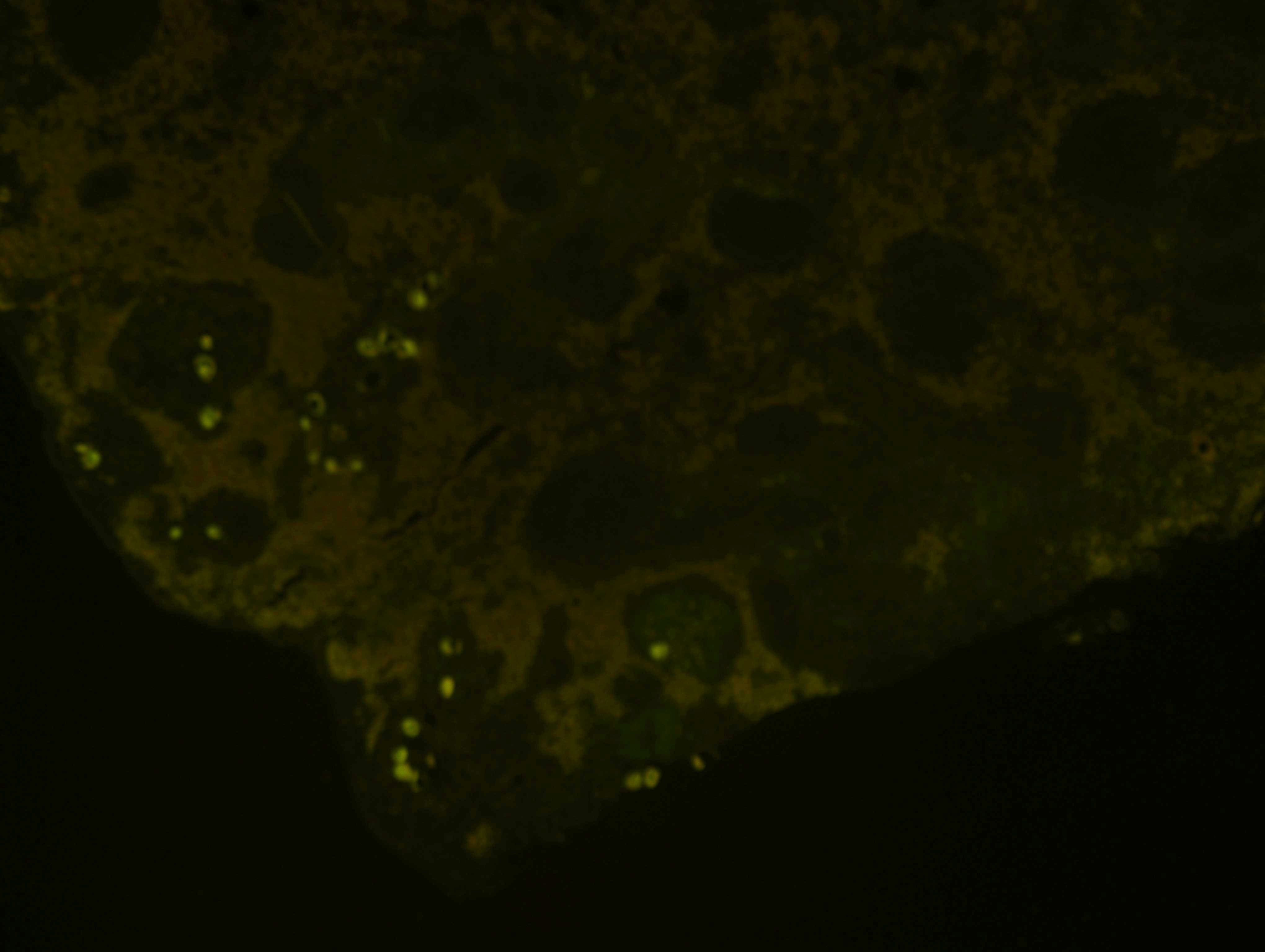

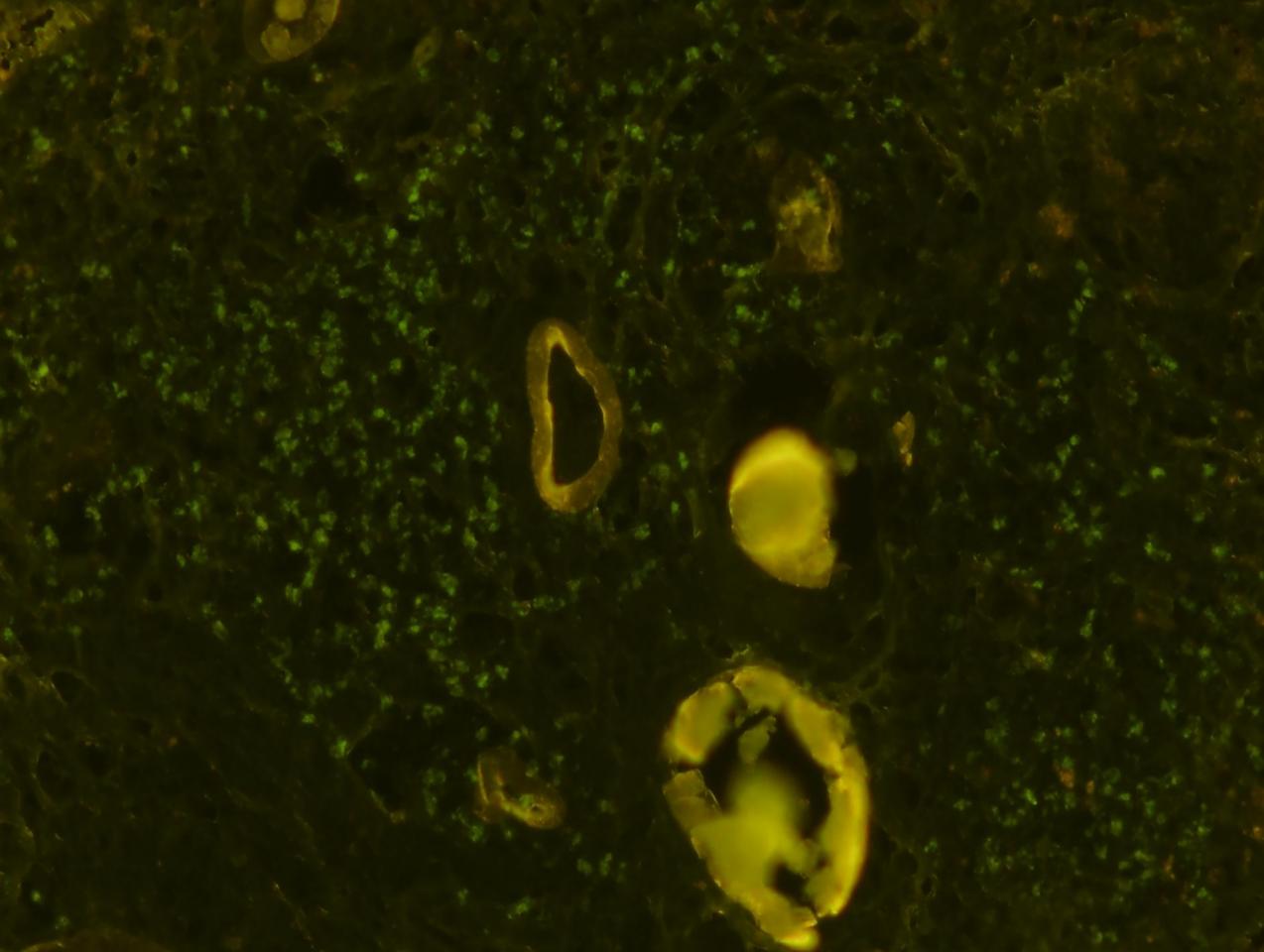
**

Figure 1E

Figure 1F

**
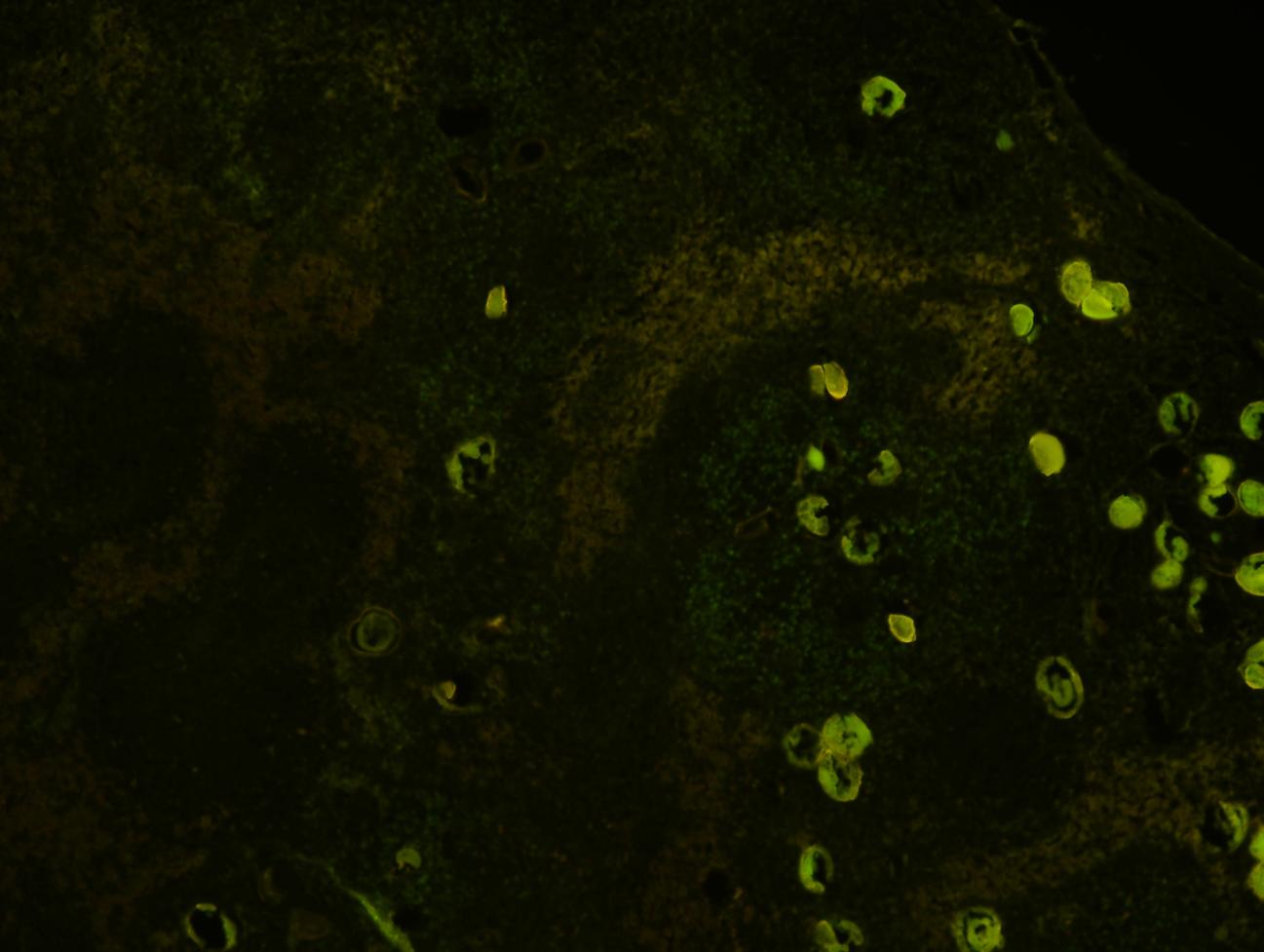
**

Figure 1G

**
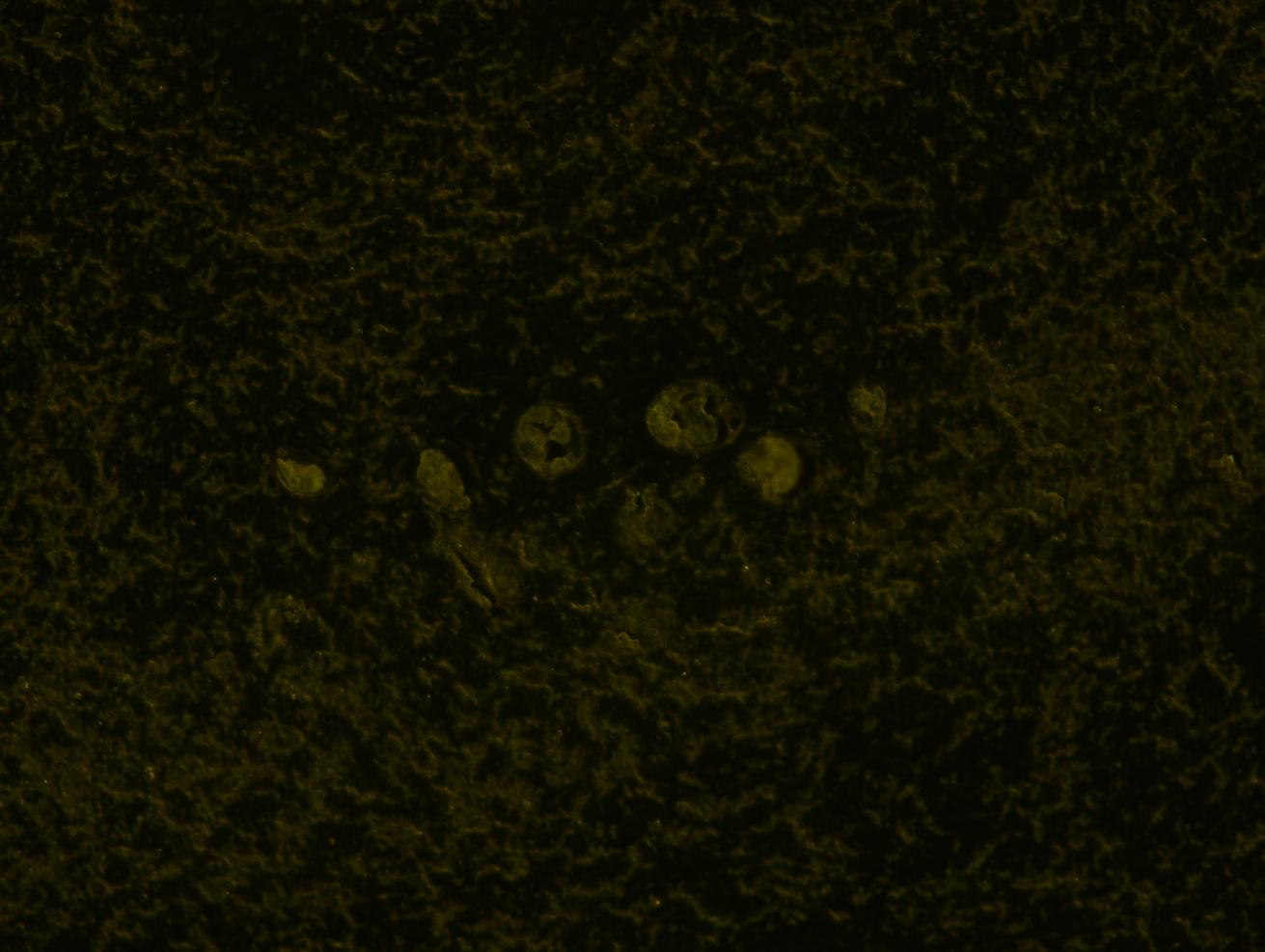
**

Figure 1H

**
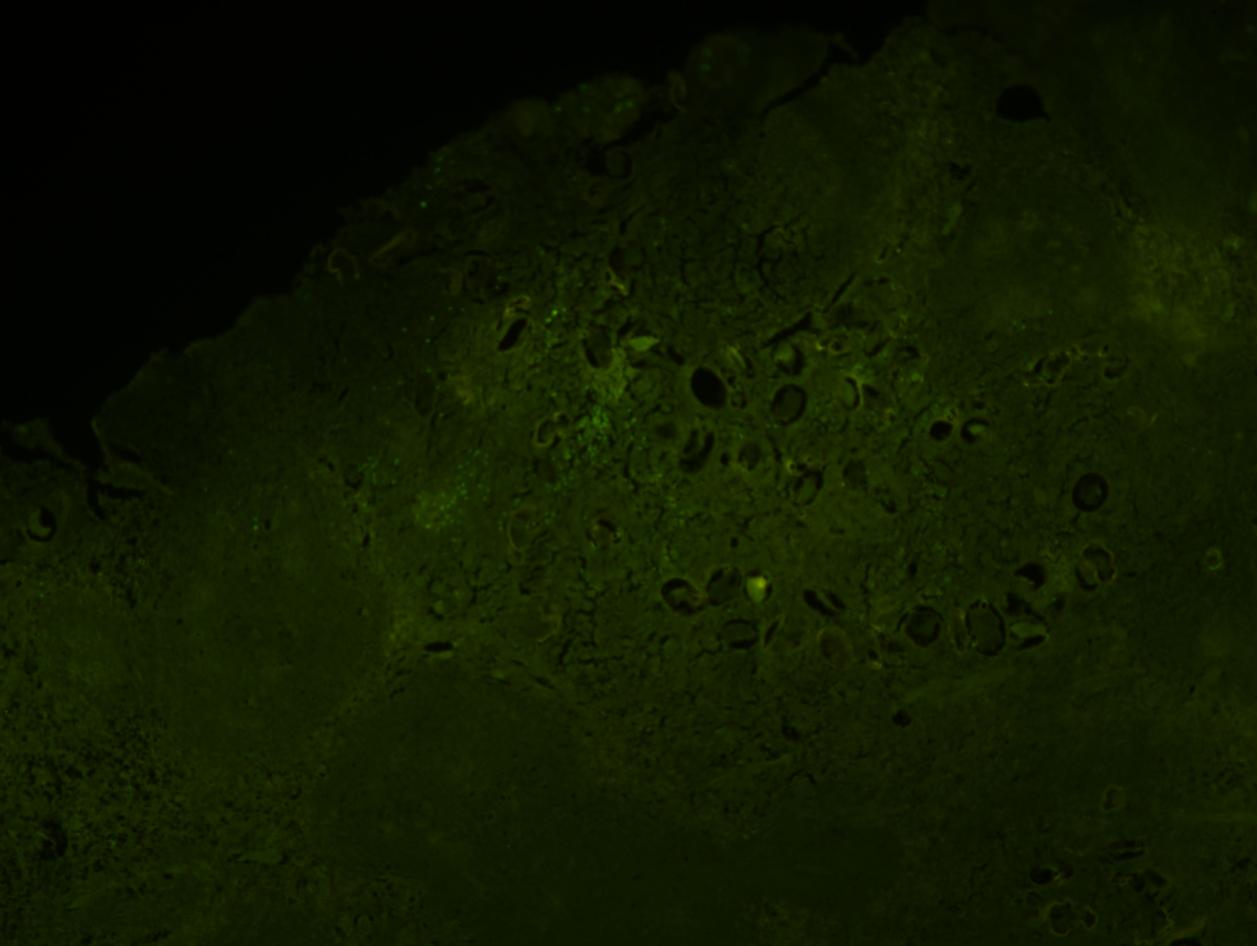
**

Figure 2A

**
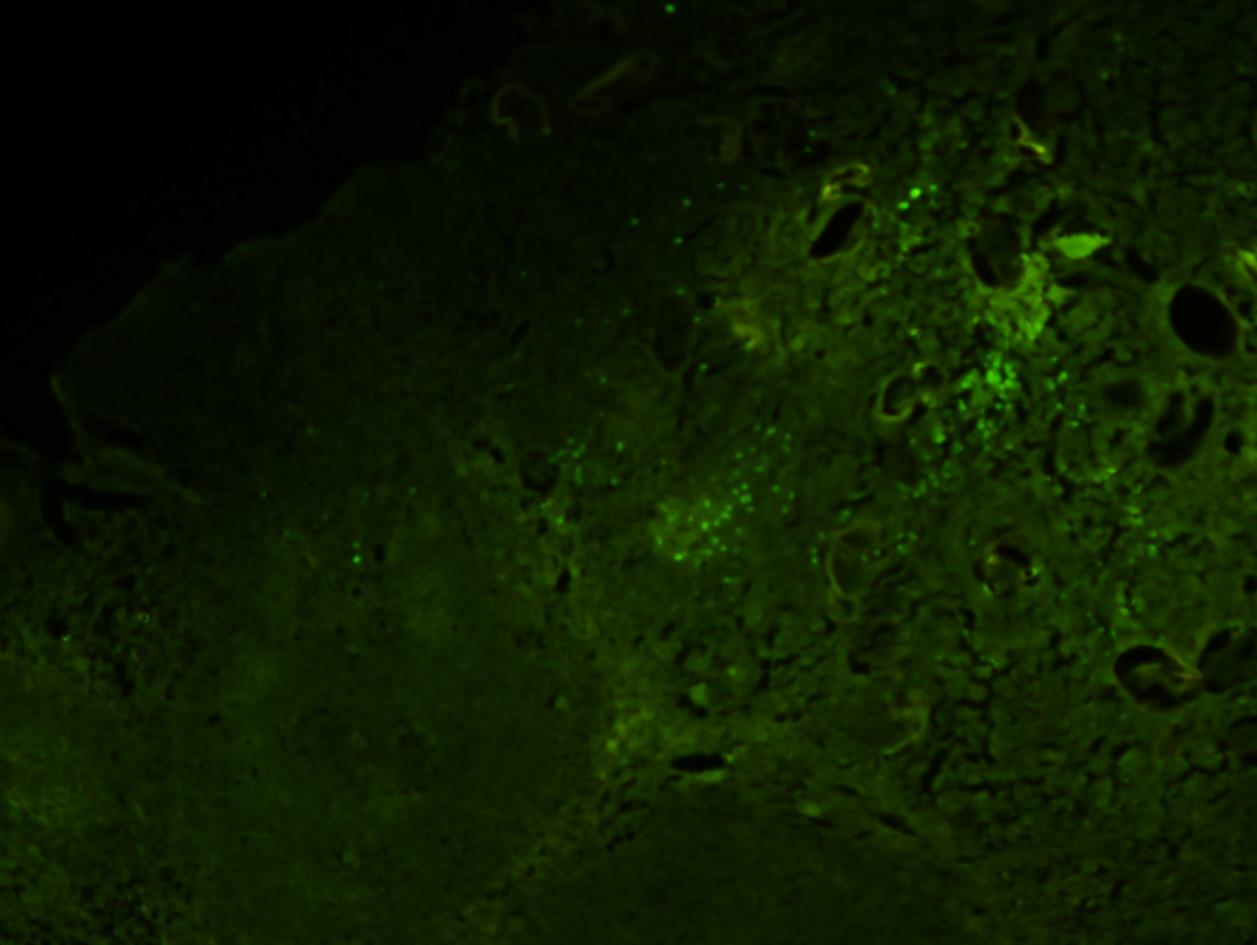
**

Figure 2B

Figure 2B


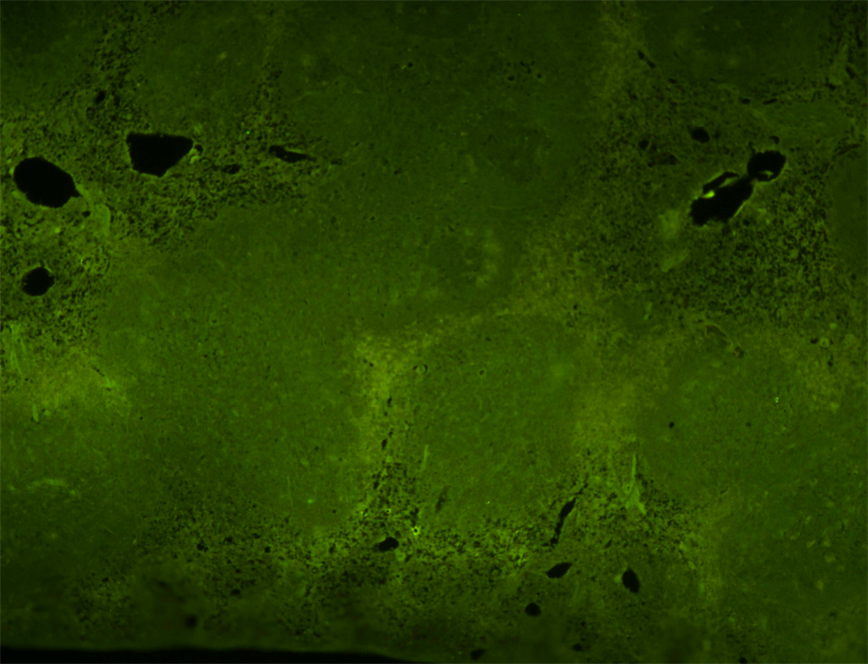

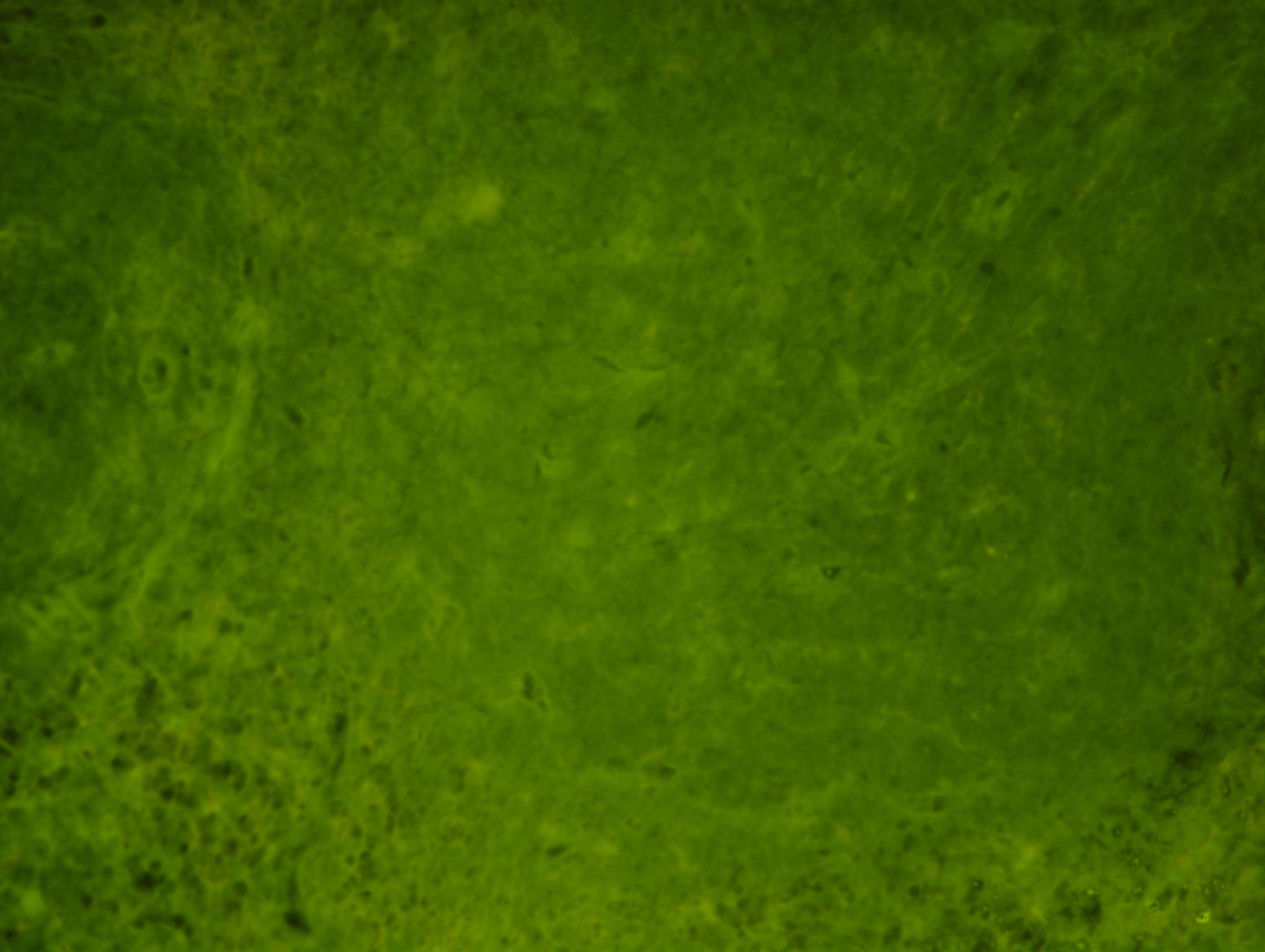


Figure 2D

Figure 2C


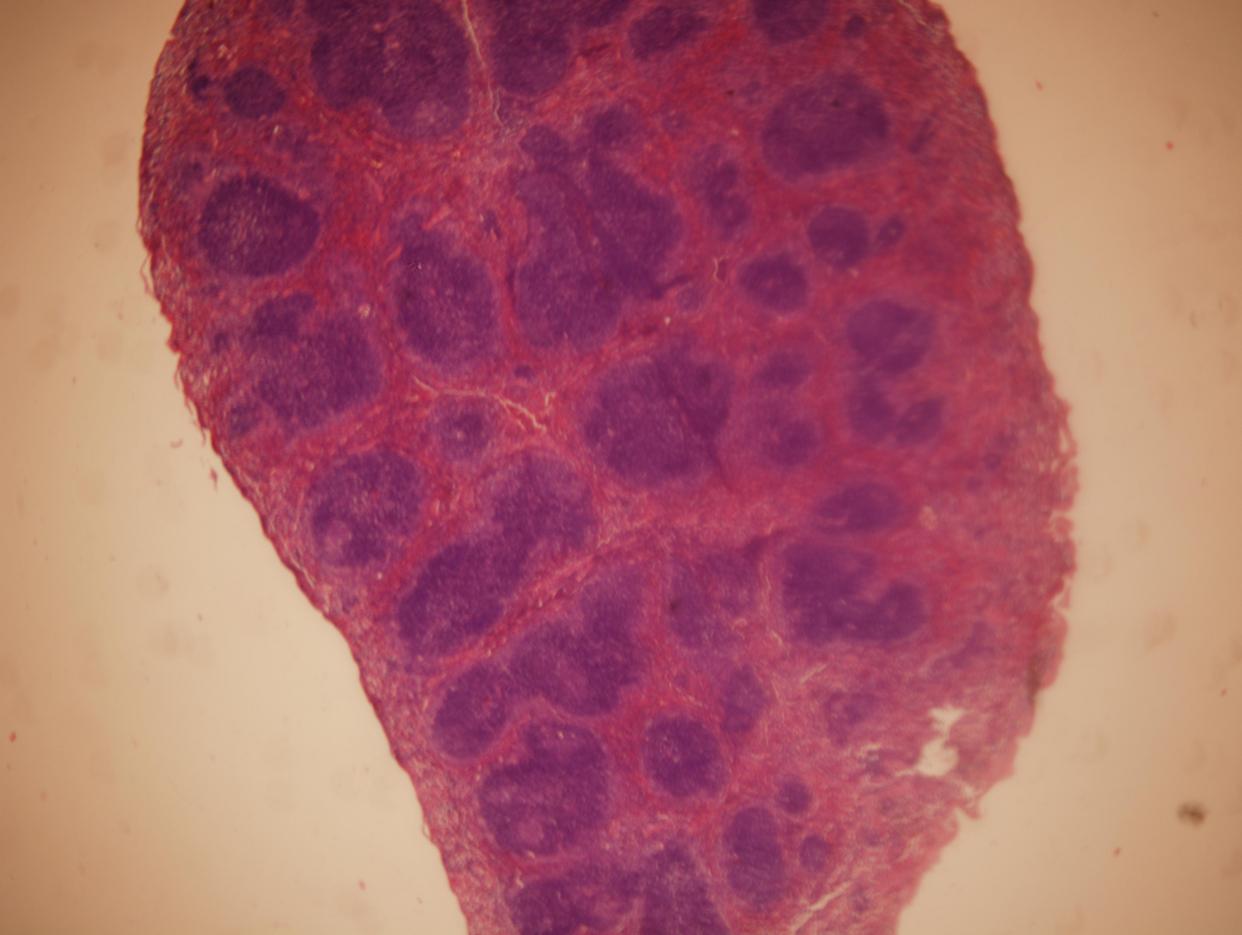


Figure 3A


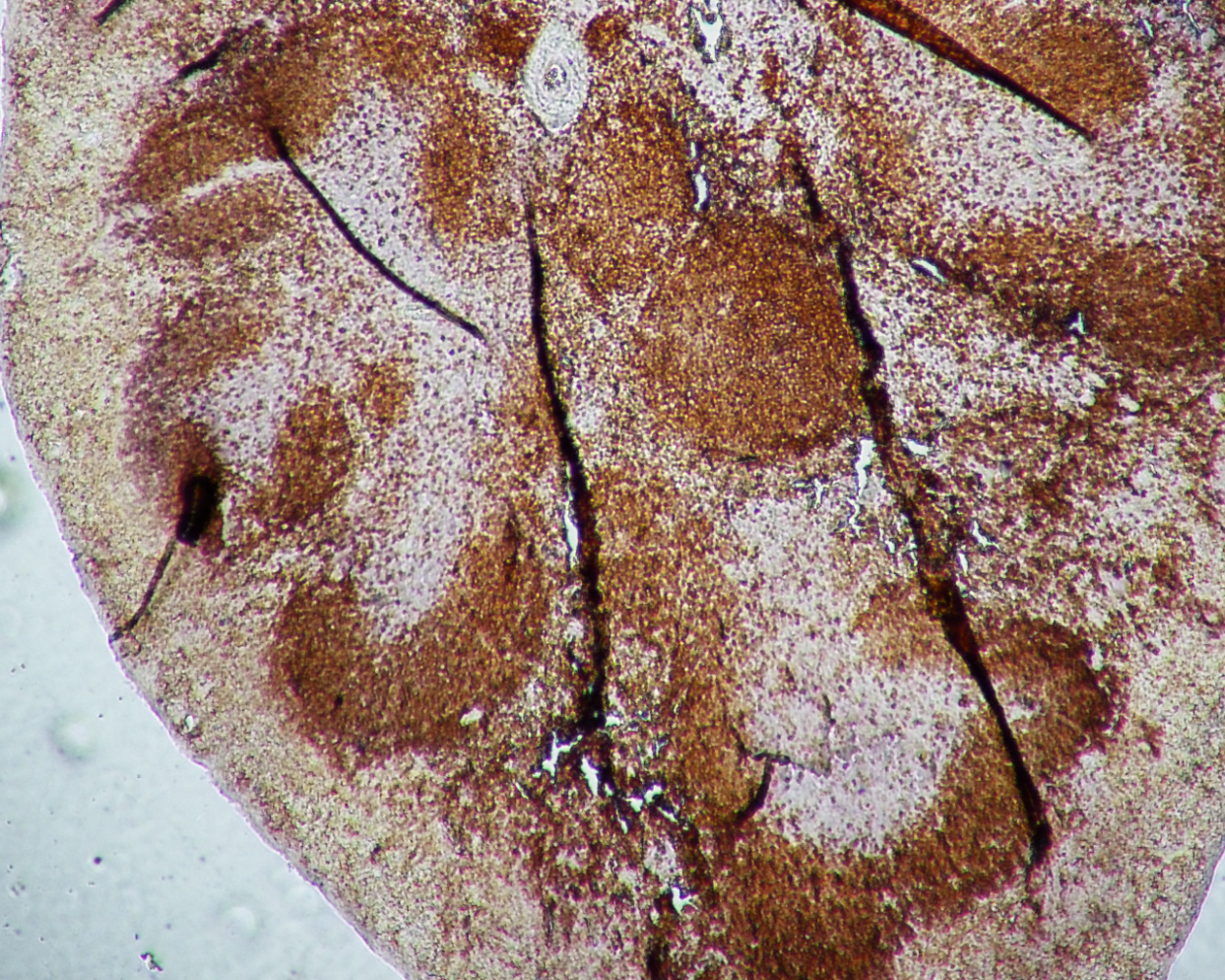

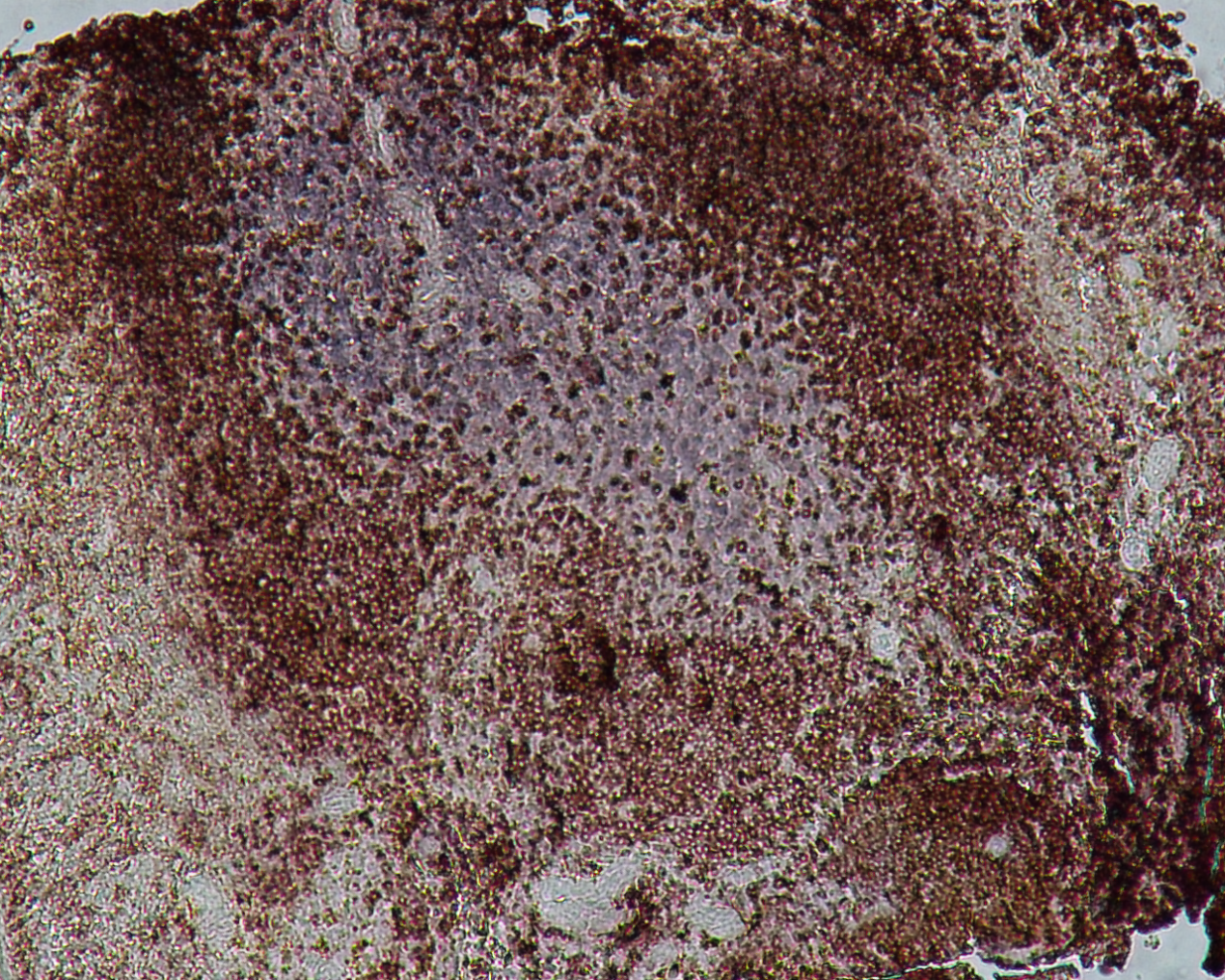


Figure 3B

Figure 3C


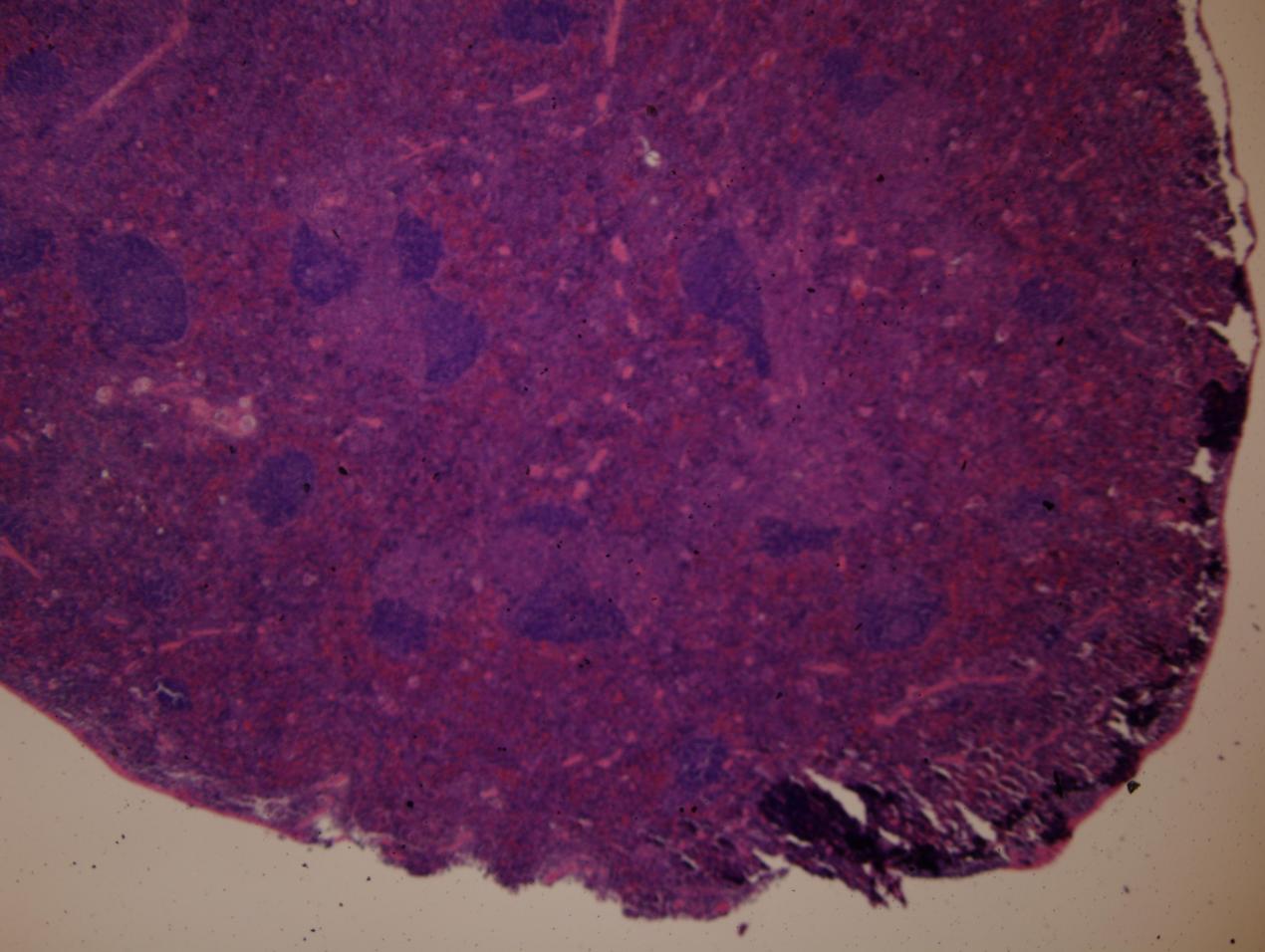


Figure 3D


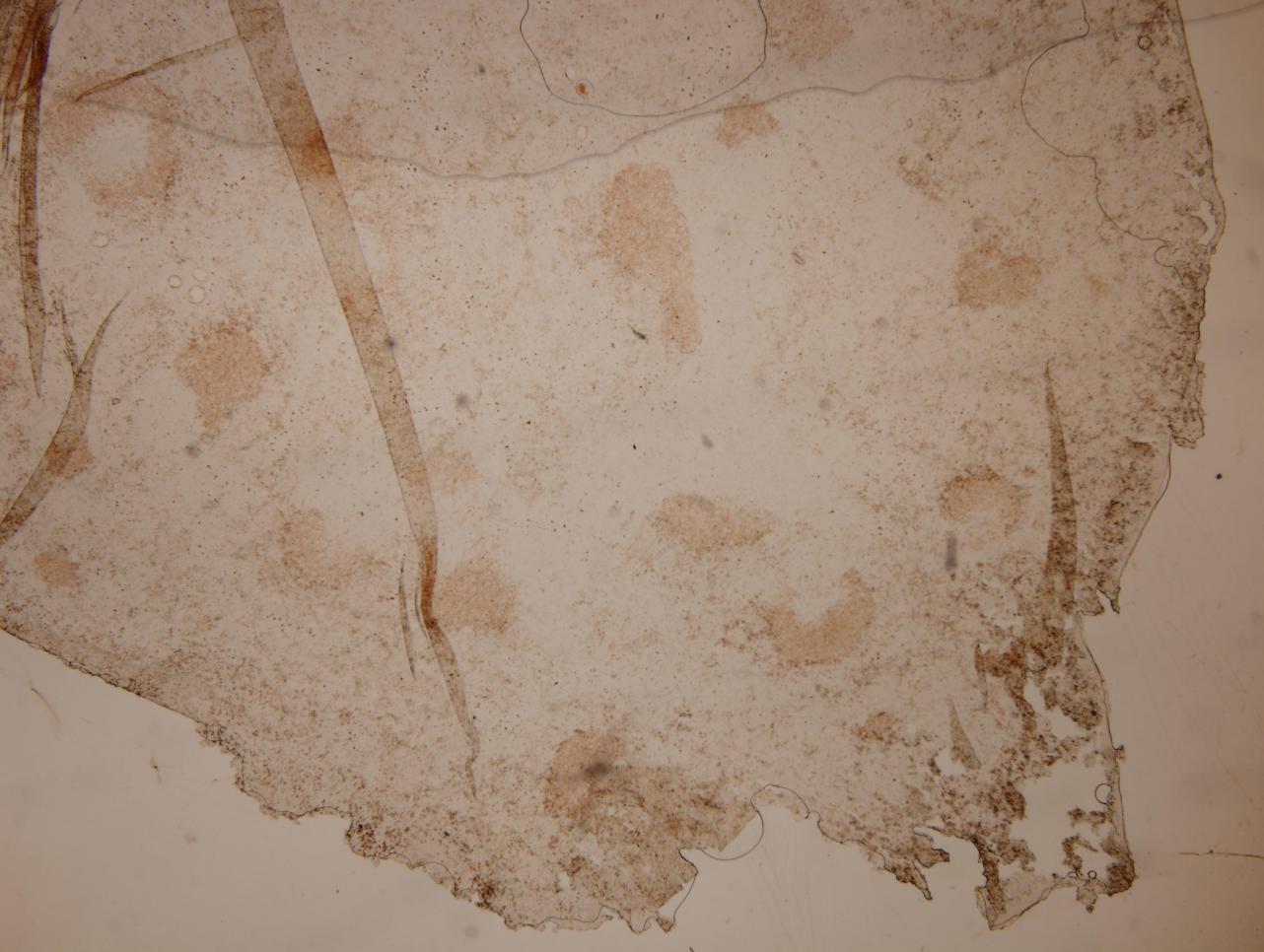

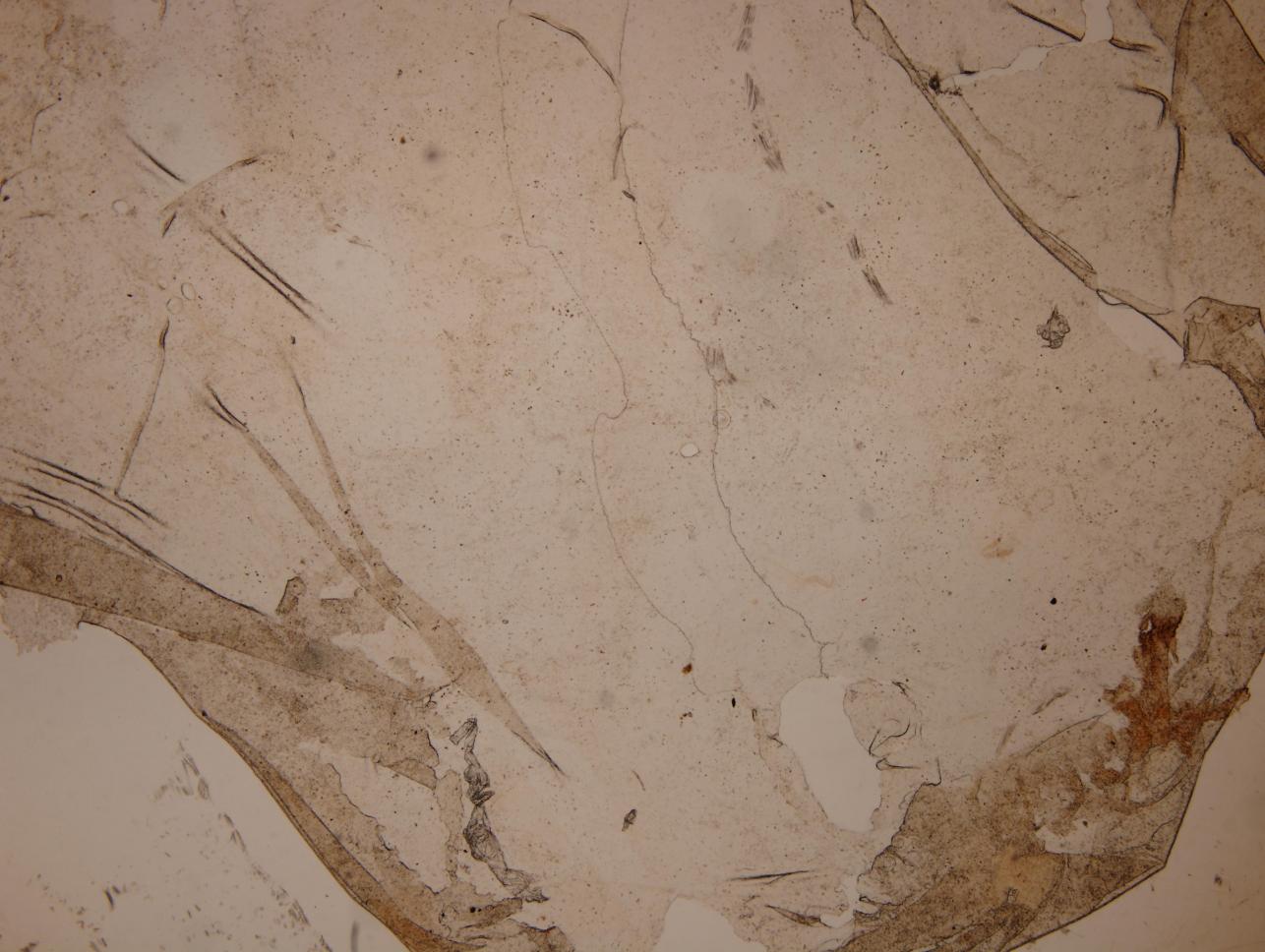


Figure 3E

Figure 3F


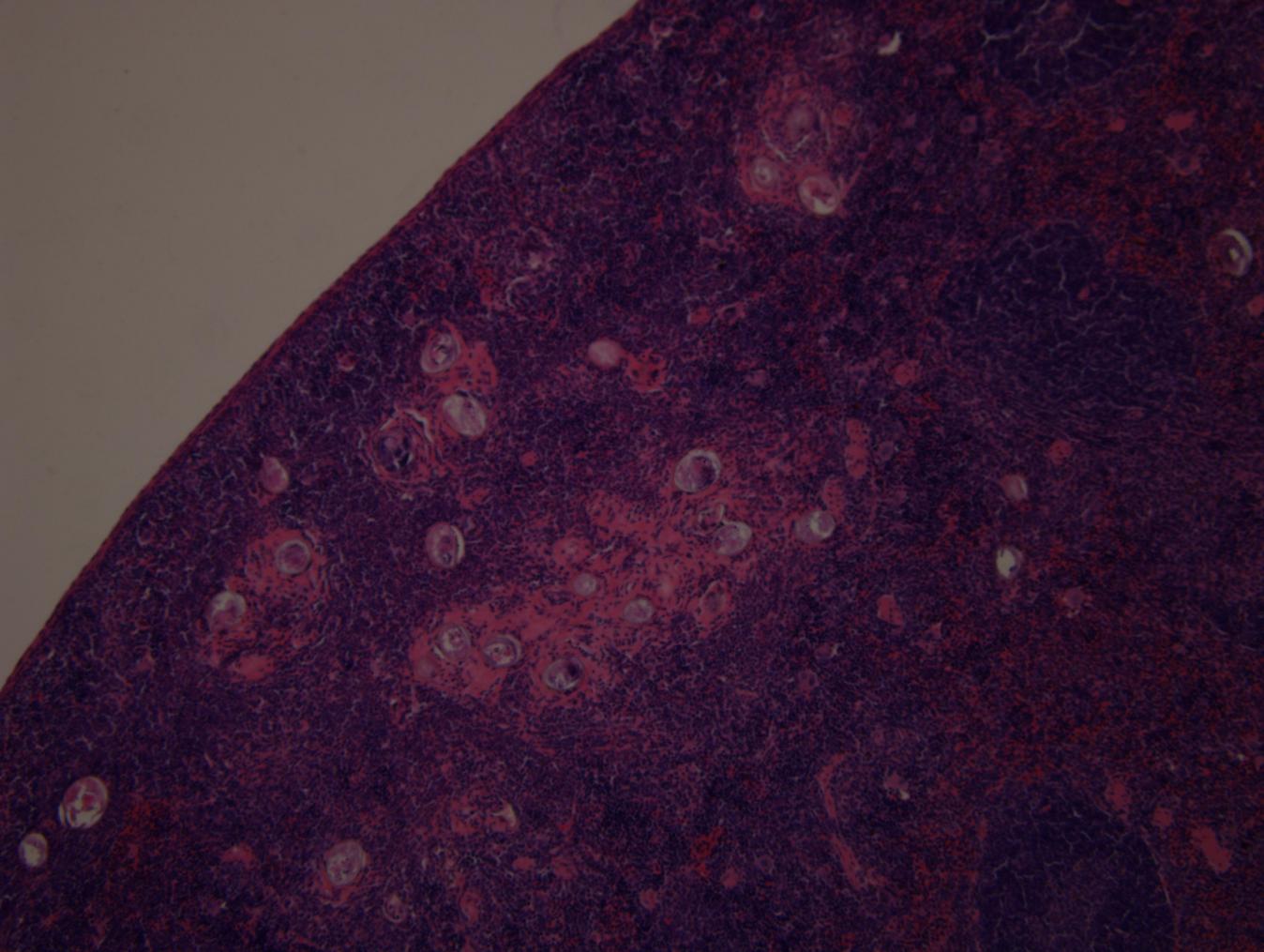


Figure 3G


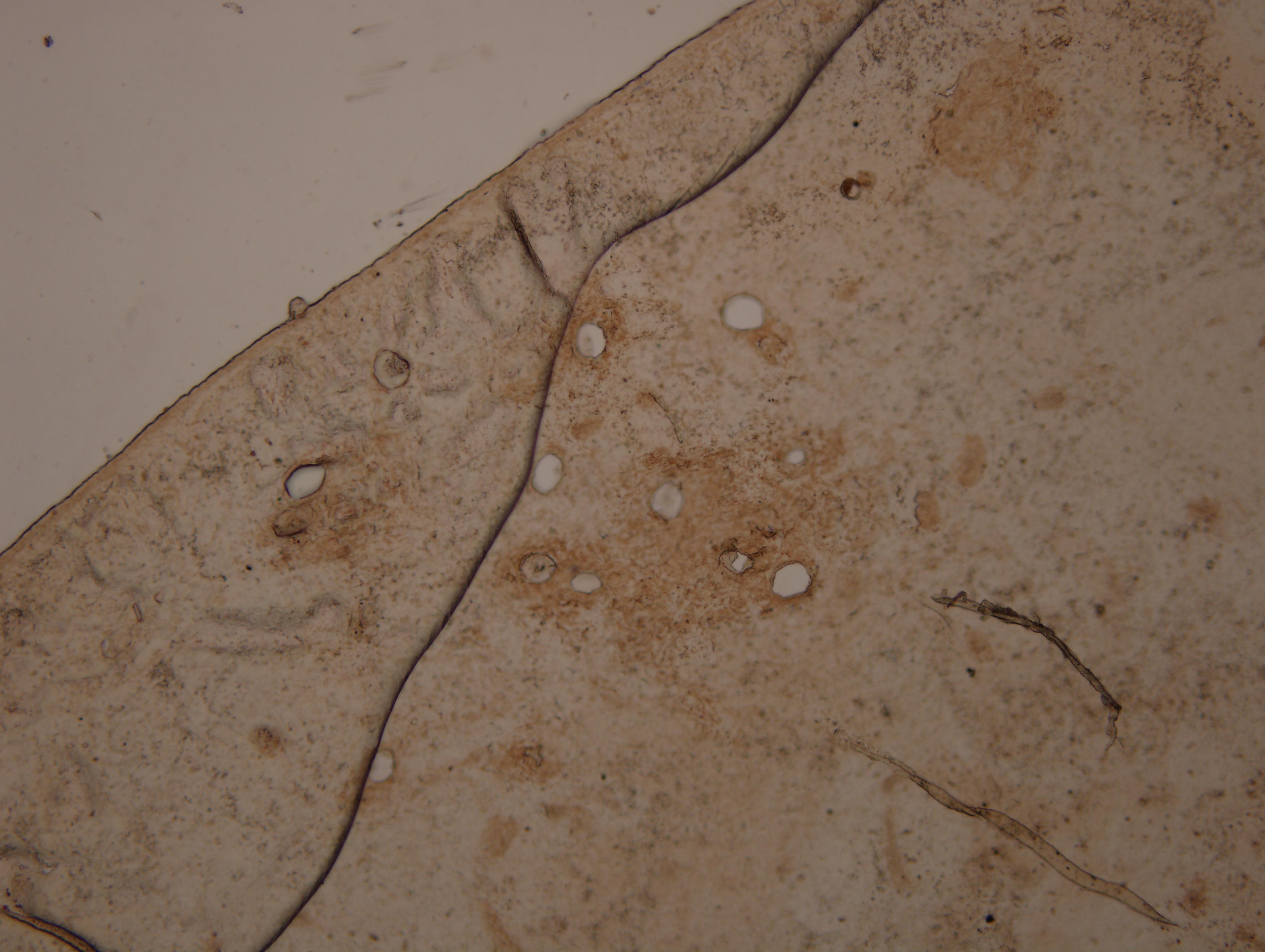


Figure 3H


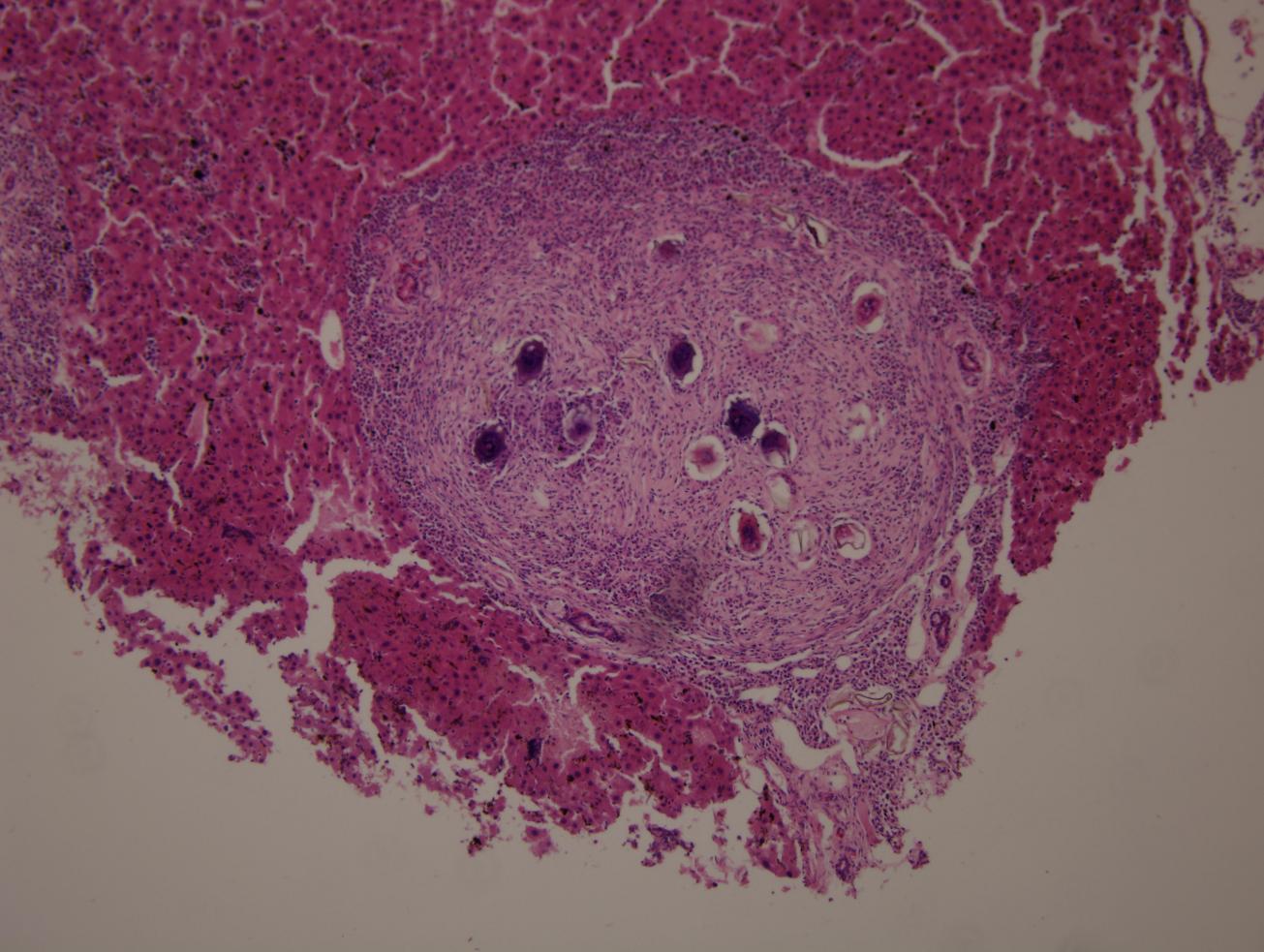


Figure 3I


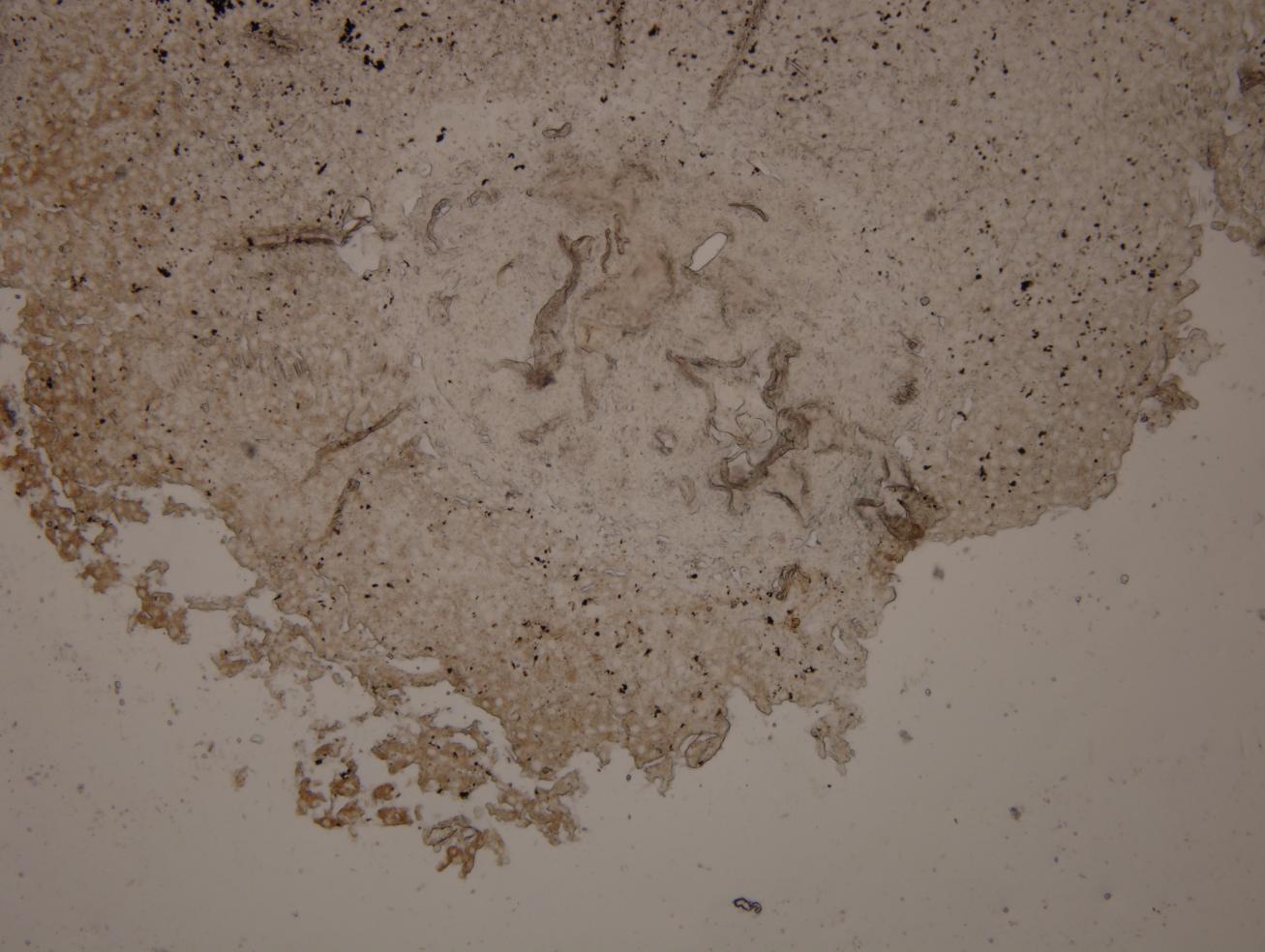


Figure 3J
