# Supplementary figures and images for: Eggs of Schistosoma japonicum deposited in the spleen induce apoptosis of splenic T cells in C57BL/6 mice
Source: Parasitol Res. 2025 Mar 10;124(3):31. doi: 10.1007/s00436-025-08474-4 (PMC11891099; doi:10.1007/s00436-025-08474-4)

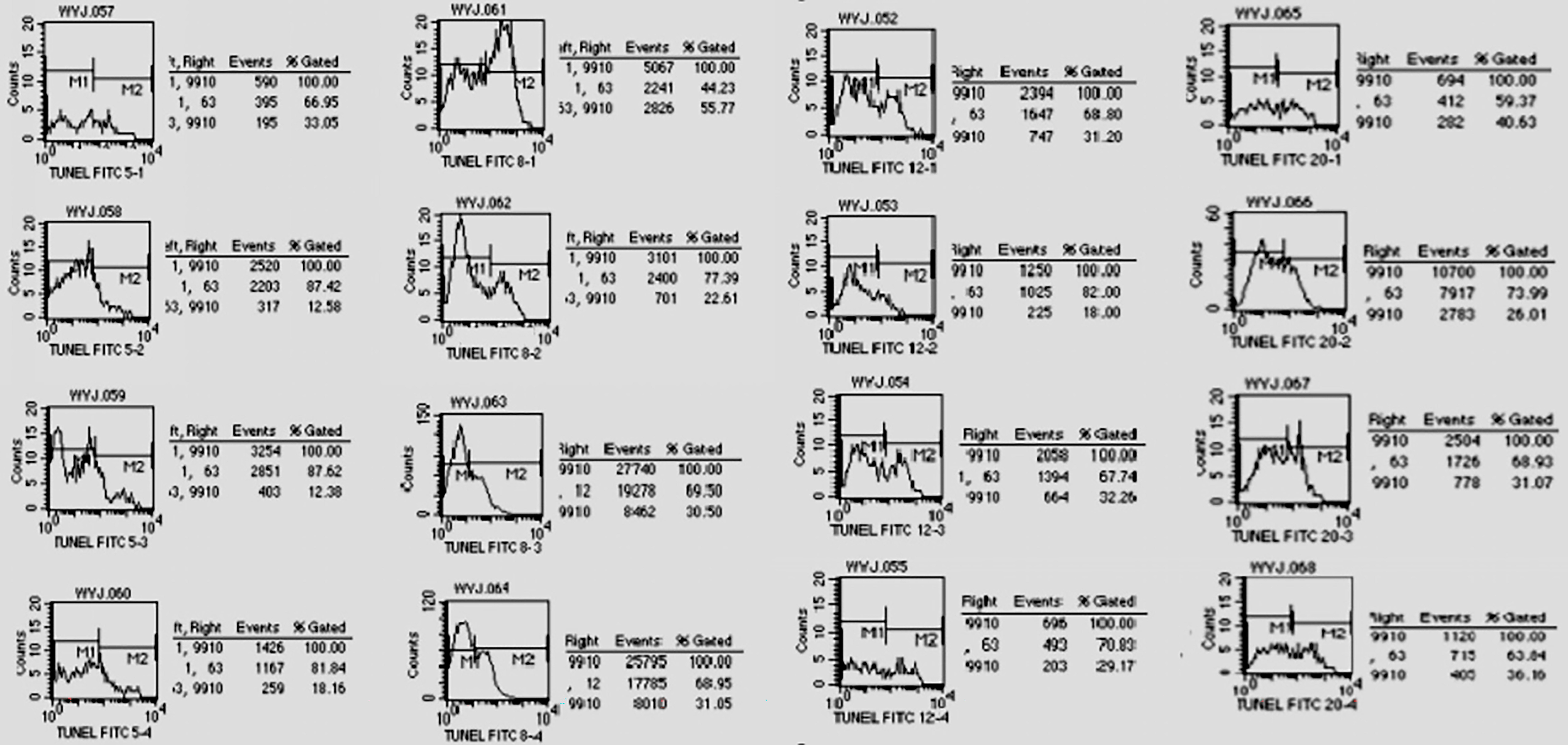


Figure 4A

Naive

40 μg/ml

60 μg/ml

120μg/ml

Supplement: Supplementary file 2 — Supplementary file2 (DOCX 2520 KB) [file 436_2025_8474_MOESM2_ESM.docx]
