# Supplementary material for: Eggs of Schistosoma japonicum deposited in the spleen induce apoptosis of splenic T cells in C57BL/6 mice
Source: Parasitol Res. 2025 Mar 10;124(3):31. doi: 10.1007/s00436-025-08474-4 (PMC11891099; doi:10.1007/s00436-025-08474-4)
Supplement: Supplementary file 3 — Supplementary file3 (DOCX 466 KB) [file 436_2025_8474_MOESM3_ESM.docx]

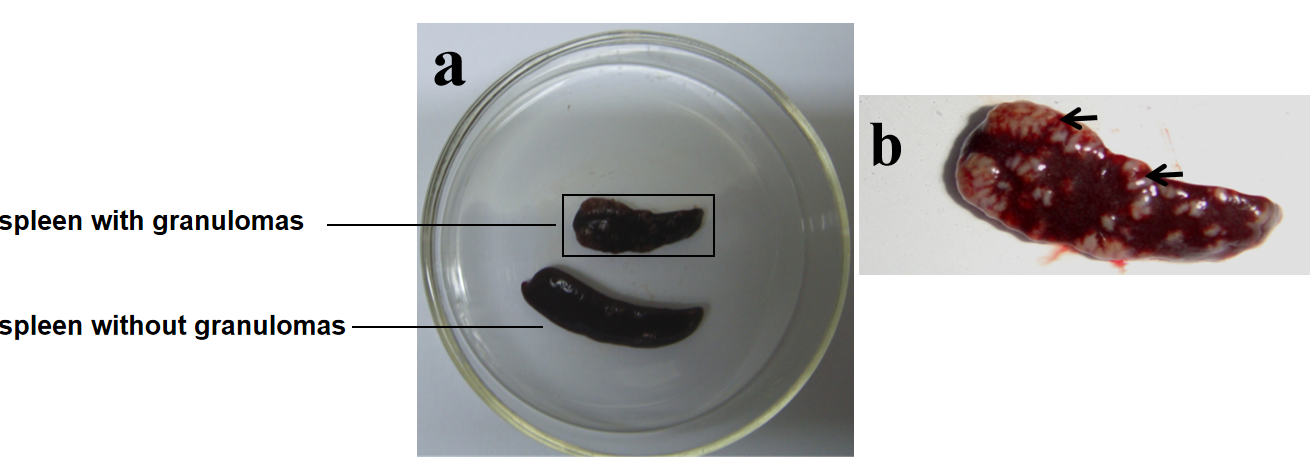


**a,** Spleens from mice infected with *S. japonicum* for 16 weeks, area in black rectangle was showed in **b**, with black arrows indicate splenic granulomas
